# Studying Socioeconomic Status: Conceptual Problems and an Alternative Path Forward

**DOI:** 10.1177/17456916221093615

**Published:** 2022-08-18

**Authors:** Stephen Antonoplis

**Affiliations:** Department of Psychology, University of California, Berkeley

**Keywords:** SES, socioeconomic position, social class, theory, measurement

## Abstract

Socioeconomic status (SES; or social class) is considered an important determinant of psychological and life outcomes. Despite this importance, how to appropriately conceive of and measure it remains unsettled. In this article, I argue that SES is, under conventional conceptions of the construct, an unmeasurable construct and present an alternative strategy for studying socioeconomic conditions. I make this argument using several lines of analysis. First, a literature review of 20 years of psychological research on SES reveals that psychologists rarely define SES theoretically (79.6% of articles did not) but call a great number of operationalizations measures of SES (147 in total). Second, current recommendations for studying SES permit contradictory predictions, rendering the recommendations unsatisfactory. Third, the appropriate measurement model for SES inhibits accumulation of results across studies, which makes studying the construct practically impossible. To rectify these issues, I reconceptualize SES as a set of socioeconomic conditions and develop a measurement strategy for studying these conditions. I conclude by considering implications for ongoing research on socioeconomic conditions and for interpreting past research on SES.

In 2016, Americans of higher socioeconomic status (SES) were more likely to vote for Donald Trump to be the next president of the United States. Higher-SES Americans were also more likely to vote for Hillary Clinton. A national survey of U.S. adults found that higher-SES individuals were more likely to report feeling stressed the day before the survey. Higher-SES individuals were also less likely to report feeling stressed (Kahneman, 2011). Finally, higher-SES individuals have tended to want greater social distance from people affected by mental illness. They have also tended to want less social distance from people affected by mental illness (Alexander & Link, 2003; Corrigan et al., 2001; Foster et al., 2018; Martin et al., 2007; Mukolo & Heflinger, 2011; Williams et al., 2018). What explains these conflicting results?

SES^1^ is considered an important determinant of psychological and life outcomes (National Academies of Sciences, Engineering, and Medicine, 2019). From health (Adler & Ostrove, 1999) to personality (Piff et al., 2010) to self-esteem (Twenge & Campbell, 2002) to stereotyping (Kraus & Keltner, 2013) to voting (Brown-Iannuzzi et al., 2015) and to psychological well-being (Tan et al., 2020), SES seems to affect a number of important psychological and life outcomes. Yet despite this importance, an answer to the question of how to study SES remains elusive (Pollak & Wolfe, 2020).

Here, I propose a solution to the issues of how to conceptualize and measure SES. I begin by describing the current state of practices for studying SES in psychology. To do so, I reviewed the psychological literature on SES and critically examined current recommendations for studying SES. After I found that current practices and recommendations do not offer good solutions for studying SES, I attempted to develop solutions using insights from psychometrics and social psychology and again found the solutions inadequate. Finally, I developed a novel conceptualization of SES and an accompanying measurement procedure to overcome the issues identified in the preceding analyses.

## How Do Psychologists Study SES?

What is the current state of how to study SES in psychology? One way to approach this question is from the perspective of a researcher beginning to study how SES affects a particular construct in which they are interested. The researcher might first look for work on how SES affects their construct of interest or similar constructs, and while reviewing the literature, might find the recommendations for studying SES. Here, I present what the researcher would learn from such a literature review and from the current recommendations.

### Literature review of current practices for studying SES

What would a researcher learn from reading the SES literature relevant to their construct? From a measurement perspective, the most important information to learn is how to make a validity argument for a measure of SES (Kane, 1992; Messick, 1989). The three key components of a validity argument for a measure of SES are: how to define SES, how to measure or operationalize it, and how to justify the chosen measure of SES. What would the psychological literature teach a researcher about this process?

#### Method

##### Identifying articles

**Fig. 1.**
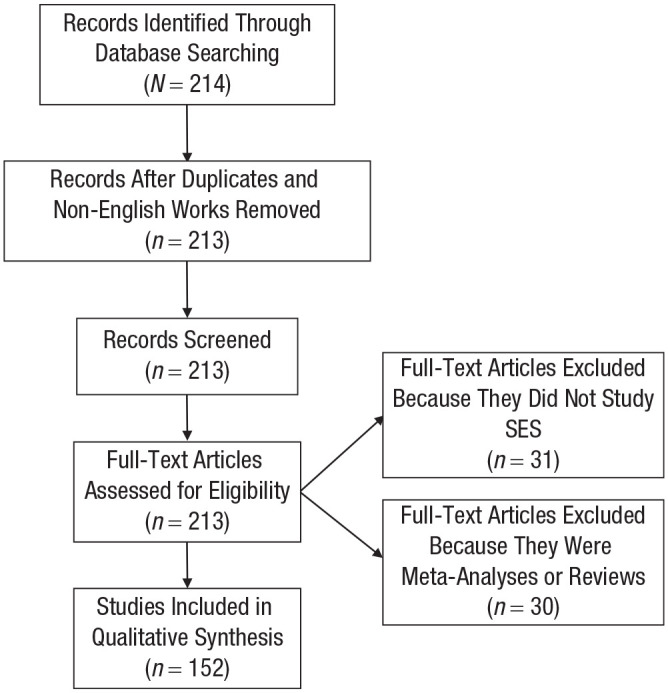
Flowchart of articles included in the literature review.

To understand how psychologists currently study SES, I obtained a set of research articles spanning many subfields of psychology and published within the last two decades. I identified articles by searching for journal articles on PsycINFO that were published between 2000 and 2019 and included one of three key phrases—“effect of socioeconomic,” “effect of social class,” and “effect of ses”—to match how a researcher might begin their literature review. Because PsycINFO includes only articles published in American Psychological Association (APA) journals, these criteria permitted me to obtain a manageably sized set of recent articles that investigated an effect of SES from a wide range of subfields of psychology (e.g., clinical, developmental, biological, social-personality). These search criteria yielded 214 articles (see Fig. 1). This procedure was closely based on the Preferred Reporting Items for Systematic Reviews and Meta-Analyses guidelines (Moher et al., 2009). The review was last updated on October 18, 2019.

I trimmed the 214 articles to remove meta-analyses (to avoid double-counting studies) and literature reviews (i.e., not empirical research; *N* = 30) and to remove non-English-language articles (*N* = 1). This yielded 183 articles for coding. Three trained research assistants then read the remaining 183 articles to extract information on whether SES was studied and, if it was, how SES was studied in each article. Through this process, an additional 31 articles were removed for not studying SES. This left 152 articles containing 224 studies for the qualitative synthesis.

##### Coding responses

Three trained research assistants extracted information on how psychologists studied SES from the final 152 articles. The following information was taken from each article: whether SES was studied as a treatment or an outcome, whether SES was studied as a main effect or an interaction, whether SES was the main interest of study, how SES was theoretically defined (e.g., “SES describes someone’s position in a social hierarchy”), how SES was operationalized, and why a particular operationalization of SES was used. Research assistants underwent extensive training on how to identify these elements of articles. I supervised, checked, and provided feedback on research assistants’ work throughout the process. I resolved any inconsistencies or ambiguities.

After all necessary information was extracted from the articles, I coded the information alone, proceeding largely inductively and developing codes on the basis of what responses occurred in the data. Some codes were adopted from prior research (i.e., Kachmar et al., 2019). I documented this process as transparently as possible (available on GitHub at https://github.com/stephanoplis/measuringSES_git). Codes categorized responses, rather than ranked them, and were not mutually exclusive. Codes were developed to categorize and provide information on how researchers thought about SES conceptually (*definition*), how they measured SES (*indicators*, *modeling procedure*), and how they justified their measure of SES (*reasons for indicators*, *reasons for modeling procedure*). From a validity-argument perspective, these correspond to how researchers intend to interpret a measure, how they intend to achieve this interpretation, and why they think this interpretation is justified (Kane, 1992). For brevity, the codes for only definitions and operationalizations are reviewed here (for review of justifications, see the Supplemental Material available online).

#### Results

##### Defining SES

How did psychologists define SES? Table 1 reports the definitions of SES used across articles. In total, 79.6% of articles did not define SES theoretically. The next most common elements of definitions were the possession of material resources (6.2%), how individuals perceive their position in a societal hierarchy (5.6%), individuals’ actual position in a societal hierarchy (2.5%), and that SES is defined by its indicators (e.g., education; 2.5%). Less common elements of definitions included SES as a system of hierarchy that ranks individuals (1.9%), a proxy for true causal mechanisms (1.2%), and a result of societal inequality (0.6%). Thus, for most articles, it was unclear how measures of SES were to be interpreted, and for the cases in which a definition was given, a variety of definitions was used.

**Table 1.** Theoretical Definitions of Socioeconomic Status

Definitions	Percentage	Number (*n* = 162)	Example
None given	79.60	129	—
Defined by indicators	2.50	4	“Class is usually defined by parental educational attainment (at least one parent with a bachelor’s or more advanced degree vs. neither parent with a degree; . . . ) or one’s own educational attainment when non-student samples are used.” (Varnum et al, 2012, p. 518)
Material resources	6.20	10	“Social class—people’s relative standing in society based on wealth and/or education” (Dubois et al., 2015, p. 437)
Perception of position in hierarchy	5.60	9	“Social class is a multidimensional construct that encompasses people’s objective resources (i.e., income, education, parental education) as well as their subjective assessments of their standing in society (e.g., subjective rank).” (Belmi et al., 2019, p. 2)
Position in hierarchy	2.50	4	“SES can be defined as a representation of an individual’s relative position in an economic-social-cultural hierarchy tied to power, prestige, and control over resources.” (Hittner et al., 2018, p. 1479)
Proxy for causal mechanisms	1.20	2	“SES is, at best, a proxy measure that in fact represents a spectrum of factors which may or may not have causal effects on reading skills or prerequisite skills.” (Corso et al., 2016, p. 34)
Result of inequality	0.60	1	“This wealth inequality yields differences in people’s relative social ranks that can be referred to by either social class or socioeconomic status (SES).” (Greitemeyer & Sagioglou, 2016, p. 178)
System of hierarchy	1.90	3	“One of the most prominent systems of hierarchy is socioeconomic status (SES), through which societies rank individuals based on their access to both symbolic and tangible resources such as wealth, education, and prestige. . . . SES is a system of stratification, in which individuals are ranked based on access to material and social resources.” (Miyamoto et al., 2018, p. 428)

Note: Percentages were obtained by dividing by the total number of codes that were applied to definitions rather than the number of definitions or studies given. Because some definitions satisfied multiple codes, the number of codes exceeds the number of studies.

##### Choice of indicators and modeling procedures

How did researchers operationalize SES? Table 2 reports the indicators and modeling procedures used across studies. Overall, many unique measurement procedures were used (147 in total), with a similarly large number of indicators (149 unique in total) and fewer modeling procedures (18 unique in total). The most common indicators were education (27.47%), income/poverty (22.83%), occupation (16.36%), and subjective status/rank (10.30%). Less common indicators included family structure (4.24%), mannerisms (e.g., clothing; 2.83%), and demographic attributes (e.g., race, gender; 0.40%). The most common modeling procedure was dimension reduction (i.e., going from multiple indicators to fewer, usually one, indicators; 40.76%), with ad hoc, less formal reduction methods the most common form of dimension reduction (e.g., sum of maternal and paternal education; 68.60%). Many studies used one (32.7%) or multiple (20.9%) single indicators of SES to measure SES; 5.2% used a mix of dimension reduction and single indicators. In sum, psychologists operationalized SES in many ways, and all researchers were able to select a measurement procedure despite most not defining the desired interpretation for the procedure’s output.

**Table 2.** Indicators and Modeling Procedures of Socioeconomic Status

Operationalizations	Percentage	Number (*n* = 495)	Example
Indicators			
None given	0.20	1	—
Assets/housing	8.10	40	Home value, own home/car, neighborhood wealth/cohesion
Composite	5.25	26	Hollingshead Index, Brazilian ABEP
Demographic	0.40	2	Race, gender
Education	27.47	136	Personal education (highest degree attained)
Family structure	4.24	21	Teen mom, father present, number of children
Income/poverty	22.83	113	Family income, neighborhood poverty rate
Mannerisms	2.83	14	Extracurricular activities, verb use, name, clothing
Occupation	16.36	81	Parental Duncan’s SEI
Subjective	10.30	51	MacArthur ladder
Uncategorized	2.02	10	Health insurance, welfare/aid, food insecurity
	Percentage	Number (*n* = 211)	Example
Modeling procedure			
None given	0.50	1	—
DR–total	40.76	86	
DR–formative	3.49	3	PCA, PLS
DR–reflective	6.98	6	EFA, CFA
DR–other composite: Readymade	20.93	18	Hollingshead Index, Duncan’s SEI
DR–other composite: Ad hoc	68.60	59	Sum of maternal and paternal education
Mixed	5.20	11	Mean of standardized income and education and income and education individually
Multiple single indicators	20.90	44	Income and education
Single indicator	32.70	69	Income or education

Note: Percentages were obtained by dividing by the total number of indicators given or by the total number of modeling procedures used (e.g., counts of indicators were divided by the total number of indicators given, *n* = 495, across all studies) rather than the total number of studies. ABEP = Associação Brasileira de Empresas de Pesquisa (Brazilian Research Enterprises Association); DR = dimension reduction; SEI = Socioeconomic Index; PCA = Principal Component Analysis; PLS = Partial Least Squares; EFA = Exploratory Factor Analysis; CFA = Confirmatory Factor Analysis.

#### Discussion

What might a researcher learn about how to study SES when starting a project on it? This research sought to answer this question via a qualitative review of how psychologists defined and measured SES in empirical articles published in APA journals from 2000 to 2019. From this review, the researcher might conclude that it is not clear how to study SES based on current practices. First, with so many articles not defining SES, it is hard to know how researchers intended their measures of SES to be interpreted and, therefore, how the interpretations of the measures should be argued to be valid. Not knowing how SES was defined makes it difficult to know how indicators of SES were chosen and how their modeling procedure was chosen, which are essential to assessing validity. Second, although it is not necessarily an issue that there were many ways to operationalize SES, the lack of definitions of SES begs the question of how the myriad measures employed should be interpreted. Their use within articles about SES suggests they should be interpreted as providing information about the same property of people. What definition of SES makes the sum of family income, parental unemployment, and whether the mother is single equivalent to the average perceived prestige of an occupation and equivalent to income and education as individual indicators? Although the empirical literature might not provide answers for how to study SES, the researcher might find the current recommendations for studying SES. Would these recommendations help?

### Current recommendations for studying SES

In the last century and a half, a variety of approaches to conceptualizing and operationalizing SES have been proposed. “Classical” approaches of the 19th and early 20th centuries, such as those of Marx (1967) and Weber (1968), focused on SES as (role-based, group-based, individual) differences in social power, prestige, and cultural and political attitudes. The “resource” treatments of the mid-20th century (e.g., Duncan, 1961; Hollingshead, 1957, 1975; Siegel, 1971) focused on income, education, and occupational prestige and how to combine these into composite indices. Somewhat later, identity, or subjective, approaches arose to emphasize the ways in which people construe their access to social and economic resources (Adler et al., 2000; Kluegel et al., 1977). With the turn of the 21st century, a new recommendation arose in psychology and public health: to avoid composite indices of SES altogether and to instead use individual indicators of SES (e.g., income, education, or occupational prestige) as measures of SES based on their theoretical relevance to outcomes of interest (APA Task Force, 2007; Braveman et al., 2005; Diemer et al., 2013; Krieger et al., 1997; Shavers, 2007). How well do the current recommendations work for creating valid measures of SES?

At first read, these recommendations seem to create valid measures of SES, as they encourage using more theoretically informed designs. Specifically, what they suggest is for researchers to operationalize SES in a more constrained way—for instance, as only income or only education. In principle, this added constraint is useful because it limits the observations that can be predicted from researchers’ theories, making falsification of competing theories more meaningful (Kellen et al., 2021). Yet a complexity arises from the fact that these recommendations treat different indicators of SES as nonexchangeable (see e.g., APA Task Force, 2007, p. 11) and at the same time want to license the interpretation of each indicator as separately measuring SES.

In particular, if indicators of SES are not exchangeable and, in fact, constitute distinct properties, then using different indicators to measure SES can produce contradictory results about how SES relates to an outcome. Researchers can conclude that SES both increases and decreases an outcome or relates and does not relate to an outcome. This issue can be seen in the findings with which I began this article. These contradictory findings were generated by measuring SES using different indicators, specifically income and education. Higher-income Americans were more likely to vote for Donald Trump,^2^ report less stress (Kahneman, 2011), and want greater social distance from people affected by mental illness (Alexander & Link, 2003; Williams et al., 2018). More educated Americans were more likely to vote for Hillary Clinton, report more stress (Kahneman, 2011), and want less social distance from people affected by mental illness (Corrigan et al., 2001; Martin et al., 2007; Mukolo & Heflinger, 2011).

What should be concluded from these results? Concluding that no effect exists does not seem appropriate because two effects do exist. Yet if the results for these individual indicators are to be understood as demonstrating effects of SES, the conclusion must be that SES does not affect voting, stress, or desired social distance from people affected by mental illness or that it both increases and decreases these outcomes. Neither of these conclusions seems satisfying. Alternatively, one might wonder whether current recommendations for measuring SES could be improved to avoid such contradictions.

## How (Not) to Conceptualize and Measure Socioeconomic Status

Given the issues described in the previous section, how should psychologists conceptualize and measure SES? Although different conceptualizations of SES are possible (see Table 1), one commonality to these and other conceptualizations is viewing SES as a unitary property of people. Building from this common understanding, two solutions have been recommended for validly measuring SES. One solution is to construct a formative measure. Another solution aims to circumvent issues in creating a formative measure and instead rely on people’s gestalt judgments of their SES (i.e., their subjective SES) to measure their (objective) SES. In this section, I argue that both of these solutions are insufficient for delivering a valid measure of SES.^3^ Instead, a different measurement procedure and conceptualization of SES are needed. I first developed a definition of SES that matches the dominant conception in the literature (i.e., as a unitary property) and then analyzed the validity of measures resulting from the two possible solutions for measuring SES.

### Defining SES

#### A definition of SES

Consistent with viewing SES as a unitary property, I adopt the following definition: SES represents individuals’ possession of normatively valued social and economic resources. Under this definition, what does a claim of identifying an effect of or on SES mean?

*Possession of* means *currently possessing*, as in *what somebody has right now (at the time of measurement)*. This definition of possession is implicit in nearly every definition and operationalization researchers use (Diemer et al., 2013). Prompts for income ask about salary *currently* or *in the last year*. Prompts for education ask about participants’ highest degree *currently* attained. Wealth prompts ask for a variety of facts, such as *current* savings or investments and debts. *Possession* covers the *status* portion of *socioeconomic status* in that it refers to a position or level. This definition centers the amount of resources individuals have, consistent with other definitions of SES (e.g., APA Task Force, 2007; Anderson et al., 2015; Kraus et al., 2012; Krieger et al., 1997) and in contrast with definitions focused on relative position.

*Normatively* means that the features of SES must always be defined for the specific populations to which researchers hope to generalize their results. If income is not a valued resource for a particular population from which a sample is drawn, then it is not a feature of SES. *Valued* means for the purpose of easing one’s (material) existence in society. That is, acquiring greater levels of SES makes it easier to comport to the “rules of the game”: paying bills, owning a house, working a good job, retiring comfortably, and so on. Thus, *normatively valued* refers to what people think is helpful for following the “rules of the game” in a particular time and place.

*Social resources* refers to human entities—usually relationships or esteem—that directly aid following the “rules of the game”. These include, for instance, prestige of occupation or of education, having social contacts who will loan one money, and having control over resources or means of production. The *directly* qualifier means that social resources that help indirectly (e.g., a secure attachment figure or a mentor at school) do not count as part of SES, helping to differentiate SES from other constructs.

*Economic resources* refers to nonhuman entities that aid following the “rules of the game”. These include, for example, wealth, income, education, and property.

Thus, SES describes how many of the human (*social*) and nonhuman (*economic*) entities (*resources*) that are commonly prized for the purpose of easing existence, or following the “rules of the game”, in a particular time and place (*normatively valued*) that a person holds (*possession of*).

Note that under this definition, SES need not be considered a real property of individuals as much as a labeling procedure created for the convenience of researchers. Although deciding whether SES is real (for general perspectives on scientific realism vs. nominalism, see e.g., Brante, 2010; Crease, 2009; Vallor, 2009) may seem tangential, adopting or rejecting realism has important consequences for deciding how to model SES (Borsboom et al., 2003; Maul, 2013). What social processes might give rise to SES? “Possession of normatively valued social and economic resources” does not seem to invoke processes of self-selection, social selection, socialization to norms, or interest-based action that give rise to occupations as homogeneous groups of individuals that can be thought of as classes (Weeden & Grusky, 2005). Instead, the construct seems to merely denote (and result from) how many resources people have. That is, SES summarizes only states of being that are related to social and economic resources, closer to a labeling function created for researchers’ convenience (Chan & Goldthorpe, 2007). Note that the view that SES results from its constituents is consistent with recent theory on the construct (Kraus et al., 2012). A nominalist account of SES thus seems appropriate.^4^

#### Advantages of this definition

This definition separates the definition and measurement of SES, unlike other possible definitions (e.g., “[SES] is usually defined by parental [or one’s own] educational attainment (“at least one parent with a bachelor’s or more advanced degree vs. neither parent with a degree . . .)”; Table 1). This separation offers three advantages.

First, it enables, in principle, broad study of SES. Separating the measurement and definition of SES allows a variety of measures to be developed, permitting broader accumulation of results (K. A. Markus & Borsboom, 2013). By contrast, if SES consists solely of one’s own or one’s parents’ education, then the 73% of studies in Table 2 not using education as an indicator did not study SES, and any of the 27% of studies using education that used additional indicators (e.g., income, wealth) also did not study SES.

Second, this definition offers a more explicit spelling out of what the construct means. In contrast, equating SES with its measurement procedure does not fully clarify what SES is. In the definition equating SES with education, the definition of education needs to be specified.

Third, this definition accords with current practices and recommendations in the field in that it permits different measures of SES to be developed and treated as exchangeable. This similarity means that analyzing this definition will be informative about the consequences for knowledge production of operating under current practices and recommendations. Psychologists treat different measures of SES as exchangeable, as evidenced by their citing research using measurement procedures different from their own to bolster the plausibility of their hypotheses. For example, the article that used the definition that equated the measurement and definition of SES (i.e., that SES is education; Varnum et al., 2012) cited an article that used subjective SES (i.e., Kraus et al., 2009) as evidence for the plausibility of its own hypothesis. Because this citation practice does not work if the measurement and definition of SES are equated, these researchers (implicitly) separated the measurement and definition of SES. Presumably, this citation practice occurs often in the literature. With the diversity of measurement procedures identified in Table 2 and elsewhere (Kachmar et al., 2019), most researchers would be hard-pressed to find articles that used the exact same procedure as them and were relevant to the hypotheses they planned to test. The current recommendations also implicitly adopt this approach in advocating the interpretation of disparate indicators as all signifying SES (APA Task Force, 2007; Braveman et al., 2005; Diemer et al., 2013; Krieger et al., 1997; Shavers, 2007).

### Measuring SES

Two solutions have been recommended for measuring SES given the theory developed in the prior section. These are the formative measurement model and subjective SES.

#### Formative measurement

**Fig. 2.**
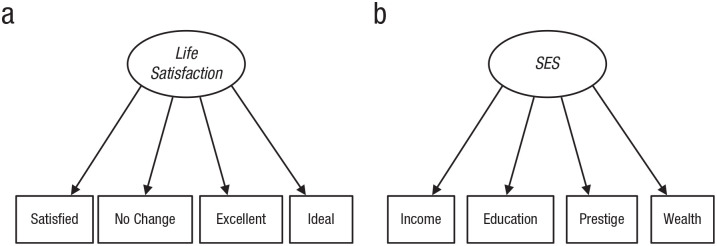
Path diagrams for reflective models of (a) life satisfaction and (b) socioeconomic status (SES).

Broadly, psychometricians have proposed two major models of latent variables—reflective and formative models. The reflective model describes a latent variable that causes variation in observed variables, or indicators (Bollen & Bauldry, 2011). Reflective models are common in psychology: For instance, being more satisfied with one’s life enables agreement with statements such as “The conditions of my life are excellent.” In the reflective model, systematic differences in responses to indicators are assumed to result from systematic differences in an unobserved, underlying attribute of participants. The causal relation between the latent variable and indicators thus flows from the latent variable to the indicators. Examples of reflective models for life satisfaction and SES are shown in Figure 2. In this example, systematic differences in SES cause individuals to have systematic differences in income, education, prestigious jobs, and wealth, just as differences in life satisfaction cause endorsement or rejection of descriptions of one’s life as good. More plainly, the model in Figure 2 asserts that people vary on an unobserved SES variable that makes them have more or less money, schooling, and prestige.

**Fig. 3.**
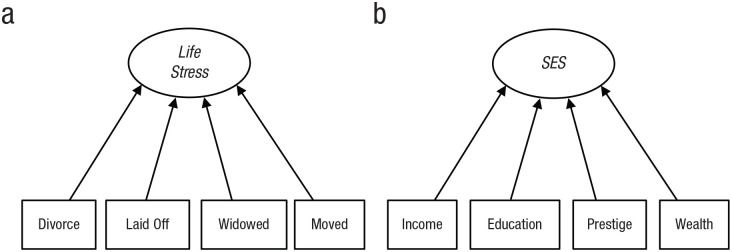
Path diagrams for formative models of (a) life stress and (b) socioeconomic status (SES).

The formative model describes a latent variable that results from variation in observed variables (Bollen & Bauldry, 2011; Bollen & Lennox, 1991; Edwards & Bagozzi, 2000). Thus, variation in the latent variable results from variation in its indicators. An example from psychology is life stress measured by stressful events such as divorce, unemployment, widowhood, and moving (see Fig. 3). Presumably, each of these indicators causes stress in people, rather than that people are already stressed and this stress increases their likelihood of being both divorced and widowed. Thus, variation in the indicators, divorce, unemployment, widowhood, and moving, causes variation in the latent variable, life stress. An example using common indicators of SES is shown in Figure 3. In this example, SES results from individuals having more or less income, education, prestigious jobs, and wealth. The model in Figure 3 asserts that people are attributed SES on the basis of their having more or less money, schooling, and prestige rather than that people are already higher- or lower-SES and then acquire more money, schooling, and prestige (as in Fig. 2).

As stated in the prior section, SES is defined as the total amount of resources and so results from its constituents rather than causes them. Any change in any indicator of SES changes SES (e.g., graduating from college), but a change in overall SES need not change all indicators of SES. Hence, the formative model is the more theoretically appropriate model to use (Edwards & Bagozzi, 2000; see also Duncan, 1992; Freedman, 1987; Pearl, 2000; Rogosa, 1987). The formative model, however, has a major problem that restricts its broad utility: It is very difficult, maybe impossible, to use with data.^5^

Formally, reflective and formative models can be stated as follows.

In the reflective model, indicator responses, *x_ij_*, for person *i* on indicator *j* result from *i*’s position on a single latent factor, η_*i*_, *j*’s loading in a factor-loading matrix, λ_*j*_, and random error, ε_*ij*_:

(1) $$x_{ij}=\lambda_{j}\eta_{i}+\epsilon_{ij}.$$

The formative model reverses this formulation so that variation in the latent factor results from variation in the indicators:

(2) $$\eta_{i}=\beta_{1}x_{i1}+\beta_{2}x_{i2}+\beta_{3}x_{i3}+\ldots +\beta_{j}x_{ij}+\delta_{i},$$

where all β_*j*_s are the effect of an *x_ij_* on η_*i*_ and δ_*i*_ is a disturbance factor, akin to ε_*ij*_ in Equation 1, representing all unmodeled indicators that contribute to η_*i*_.

The principal drawback of formative models is that estimating the effect of indicators on the latent variable often leads to interpretive issues. In reflective models, estimating paths from the latent variable to indicators is easier by virtue of factor analysis. Factor analysis assumes that a latent variable (e.g., life satisfaction) causes all of its indicators, with this common causation inducing covariation between indicators (e.g., as represented in the correlations between indicators from the Satisfaction with Life scale). The paths from the latent variable to indicators are then estimated as those factor loadings that reproduce the covariance matrix of indicators as closely as possible. In formative models, indicators are not assumed to have any common cause but to have a common outcome. Thus, factor analysis cannot be used to estimate the paths from the indicators to the latent variable. Instead, the formative latent variable must be estimated first because it is not possible to estimate a causal effect on a variable that does not exist. How can researchers estimate the latent variable in the formative model?

**Fig. 4.**
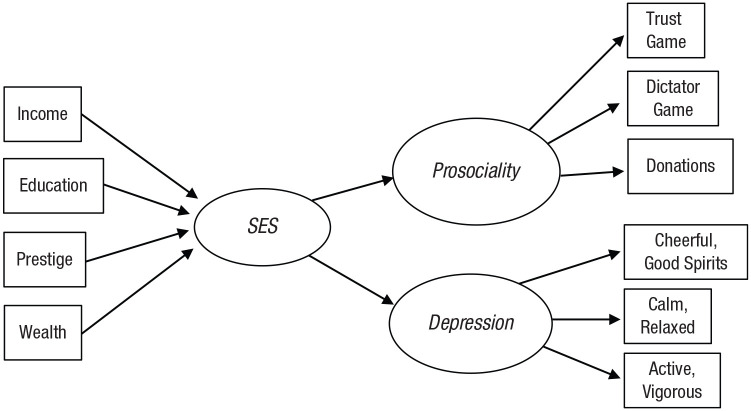
Illustration of a formative model for socioeconomic status (SES) with two reflectively measured outcomes.

Figure 4 shows an example of how to estimate SES as the latent variable in a formative model. It works by embedding the measurement model for SES in a larger structural model. In Figure 4, SES is formatively measured by income, education, occupational prestige, and wealth; in turn, SES causes prosociality and depression, each of which is reflectively measured by three indicators. To estimate SES, researchers can notice that in the same way that prosociality and depression are reflectively measured by their indicators, SES is reflectively measured by prosociality and depression. Researchers can then represent SES as the shared variance of prosociality and depression and estimate how income, education, occupational prestige, and wealth relate to this shared variance. Hence, all the paths in the model can be estimated. What about this process yields interpretive issues?

There are three problems with this approach, and they all relate to the validity of interpreting the procedure’s output. First is interpretational confounding, which occurs whenever a construct’s meaning differs from the researcher’s intended meaning (Burt, 1976; see also Duncan, 1992; Freedman, 1987; Rogosa, 1987). Interpretational confounding can occur in two ways with formative models. First, the shared variance of the outcomes that researchers are interested in need not correspond to the meaning of SES (K. A. Markus, 2014; Rhemtulla et al., 2015), and second, it likely varies across studies, depending on which outcomes are studied (Howell, 2014; Howell et al., 2007). Within a single study, the shared variance of SES’s outcomes need not correspond to the definition of SES. For example, does the shared variance of prosociality and depression capture how many normatively valued social and economic resources someone has? Between studies, different sets of outcomes produce different empirical meanings of SES while the formatively measured latent variable remains labeled as SES across studies. For instance, if Figure 4 were altered so that the outcomes were more conceptually similar (e.g., depression with satisfaction with life, prosociality with antisocial behavior), one alteration would produce an estimate of SES consisting of variance related to psychological well-being (depression and satisfaction with life) and the other related to helpful/harmful behavior (prosociality and antisocial behavior). Because well-being and helpfulness are not the same constructs, these two alterations do not produce equivalent estimates of SES.

Second, unless researchers are simply studying tautologies (i.e., the outcomes are all variables that could be called indicators of SES), the structural model is unlikely to fit the data well. Bainter and Bollen (2014) showed that a structural model involving a formatively measured variable fits well only when all possible outcomes of the formative variable result solely, or primarily, from the formative variable (i.e., exhibit unidimensionality with respect to the formative variable). In other words, the model displayed in Figure 4 will fit data well only when prosociality and depression result solely, or primarily, from SES. This (strong) criterion seems unlikely to be met in most research on SES.

Third, in formative models, unlike in reflective models, indicators are not exchangeable (Bainter & Bollen, 2015; Bollen & Bauldry, 2011). Thus, using different sets of indicators to formatively model the same latent variable does not actually produce the same latent variable. For instance, if Figure 4 were modified so that only income and education or only occupational prestige and wealth were used to measure SES, the resulting models would not be equivalent in meaning because each set of indicators predicts different portions of the shared variance of prosociality and depression, producing different formative variables. Ignoring this can yield a situation in which an apparent effect of SES measured by only income can “fail to replicate” when measuring SES using only education. Claiming “failure to replicate” in such a situation does not make sense because education and income are not exchangeable constructs. Thus, calling the effects of income and education separate effects rather than subsuming both under SES may be a better strategy. In sum, formative measurement does not provide a valid measure of SES.

#### Subjective SES

Subjective SES has been proposed as a remedy to the problem of deciding which indicators of SES to include in a formative model. In particular, subjective SES—typically measured by the MacArthur ladder or self-identified categorical rank (i.e., lower class, working class, middle class)—is thought to provide an aggregate measure of objective SES (Adler et al., 2000; Adler & Stewart, 2007; APA Task Force, 2007). The promise of this construct is that by being subjective, it permits participants to aggregate all the relevant information for assessing SES into a single point score for researchers, thereby avoiding the problem of trying to account for all important aspects of SES when measuring it. Moreover, it permits participants to account for variables that researchers typically do not measure, such as school prestige. Finally, it relies on participants’ broader conceptions of themselves as high, middle, or lower class, permitting the use of a reflective measurement model instead of a formative model.

Although appealing, the expectation that subjective SES provides efficient aggregates of objective indicators of SES is odd for a few reasons. First, given that psychologists struggle to define SES conceptually and in terms of objective indicators, it is not clear why research participants (rushing through a survey) should be able to aggregate every objective indicator of SES in their heads and transfer that aggregate to a 10-point ladder metaphor (or 5-point category scheme). Second, even if participants perform this aggregation, there is a validation problem: If researchers lack a measure of SES in terms of only objective indicators, what could be used to determine whether the subjective measures capture an aggregate of the objective indicators? Third, the subjective aspect of subjective SES means participants can include information in their ratings that researchers do not want included. For instance, in an unpublished qualitative study of how participants determine their MacArthur ladder position, researchers found that in addition to expected aspects such as material wealth, occupation, and education, participants used their spirituality and ethical values, prosocial behavior, and health to determine their MacArthur ladder ratings (Adler & Stewart, 2007). The inclusion of the latter three attributes poses a serious problem for researchers hoping to study the causal effect of subjective SES on values, prosocial behavior, or health outcomes because these variables overlap in meaning with subjective SES (Edwards & Bagozzi, 2000). Fourth, the subjective aspect also means that participants can aggregate information about their conditions in different ways. For instance, if researchers wanted to interpret measures of subjective SES as direct readouts of people’s social and material conditions, they would not expect Black Americans to have higher subjective SES ratings, on average, than White Americans because of existing racial inequalities in the United States. Yet Black Americans do rate themselves higher than White Americans on subjective SES (as the MacArthur ladder) despite scoring lower on every objective indicator of SES measured (i.e., education, employment, income; Shaked et al., 2016). In sum, subjective SES also does not appear to offer a valid measure of SES.^6^

## A Novel Solution: Studying Socioeconomic Conditions

Given the issues described, how should researchers study SES? The preceding analysis suggests an initial answer: to reconceptualize SES as a set of structural features and to study the individual features traditionally thought of as indicators of SES as their own constructs.

Under this conceptualization of SES, researchers would think of SES as a set of structural features that guide the decisions and behaviors people take instead of as a unitary property that they try to measure and study effects of. Instead of saying that SES causes outcomes, researchers would say that the structural features SES contains (e.g., income, education) cause outcomes. In the same way that race and gender are structural features of people’s environments but do not measure the same property (i.e., “structural location”; Halasz & Kaufman, 2020), researchers can say that traditional indicators of SES (e.g., income, education) are distinct structural features of people’s environments that do not measure the same property (i.e., SES). Instead, these socioeconomic conditions are a sequence of achievements and acquisitions that make further achievements and acquisitions more likely to occur. That people are college-educated makes it easier for them to have well-off friends who might share advice on career and financial opportunities that make building wealth easier. If researchers reconceptualize SES as a set of interacting conditions, the question of how to study SES no longer focuses on developing a valid measure of it. Instead, studying SES consists of choosing structural features contained in SES as the features are relevant to outcomes under investigation. Thus, researchers would study the effect of income or education (or their interaction) on an outcome rather than measuring either (or both and aggregating them) and inferring an effect of SES. Researchers would infer only an effect of income or education (or their interaction).

This conceptualization of SES is consistent with several positions developed earlier. First, consistent with the nominalist view of SES, SES need not be thought of as real if it is regarded as a set of properties but not a property itself. Instead, it is a helpful organizational concept that points to similar structural properties researchers might consider when explaining phenomena. For example, income and education are similar in that they both help people follow the “rules of the game”: Having more money makes paying bills and acquiring property easier; having more education makes acquiring higher-income jobs easier. Yet they need not make people’s lives easier in similar ways: Income is often thought of as easing the purchase of valued commodities (e.g., health care; Shavers, 2007); education is often regarded as providing cultural fit advantages in certain contexts (e.g., H. R. Markus & Kitayama, 2010; Stephens et al., 2007). The concept of SES helps remind researchers that these properties might help explain the phenomena they care about, even if they do not claim that studying income or education reveals an effect of SES.

Second, this approach is consistent with various positions advocating the use of theoretically informed measurement procedures. Changing the question of “How do researchers study SES?” from “How do researchers measure SES?” to “Which feature of SES should researchers study?” points researchers toward more theoretically informed measurement strategies. This is consistent with the validity argument’s goal of identifying measurement procedures that yield desired interpretations (Kane, 1992). It also fits with the position from sociology to rely on theoretically driven models over statistical machinations (Duncan, 1992; Xie, 2007). Finally, in measurement science, Wilson and Gochyyev (2020) argued that when testing scientific theory is the goal, it is more informative to use the indicators creating formative variables than to use the formative variables themselves.

Below, I describe a decision tree to help researchers select features contained in SES that fit researchers’ theoretical aims. I also demonstrate how to use the decision tree. Throughout, I use the label “socioeconomic conditions” instead of “socioeconomic status” or “SES” because the former more clearly suggests the multiplicity of features referred to by the concept, whereas the latter two labels imply a certain thingness, unity, or wholeness that does not match the conceptualization of SES used in the decision tree.

### Measuring socioeconomic conditions

**Fig. 5.**
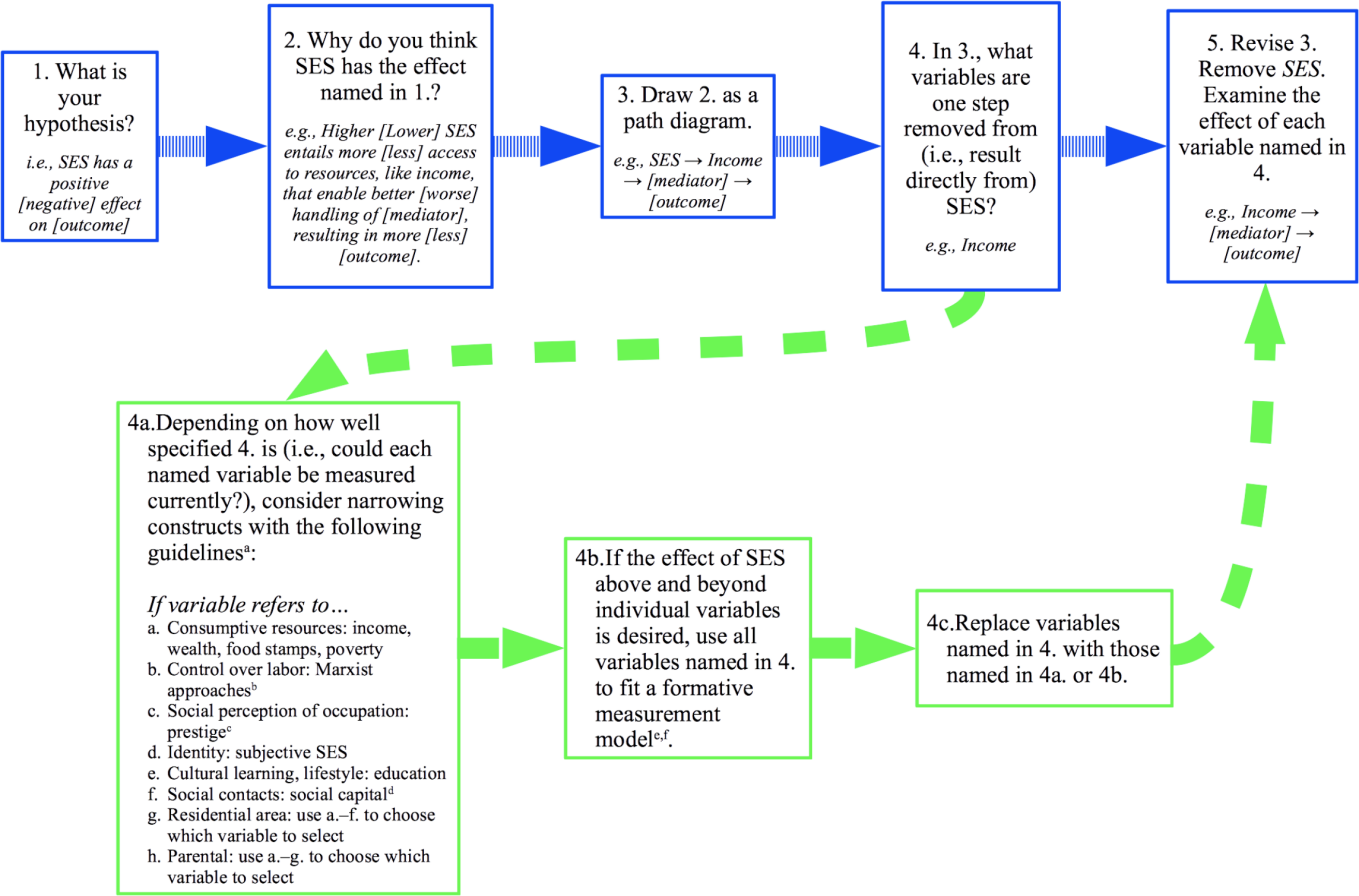
Decision tree for measuring socioeconomic conditions. ^a^Diemer et al. (2013) provided an excellent set of pragmatic considerations when measuring many of these variables. Galobardes et al. (2006a, 2006b), Krieger et al. (1997), and Shavers (2007) provided a description of theoretical strengths and limitations of income, wealth, education, and other socioeconomic conditions. ^b^For example, Wright (1997) and Wright & Perrone (1977). ^c^See Haug (1977) for very serious concerns about the validity of existing prestige measures. ^d^See Coleman (1988) for a theoretical discussion of social capital. Tulin et al. (2018) provided one example of measuring social capital. ^e^Note that procedures for selecting indicators for formative models are largely undeveloped (West & Grimm, 2014). Diamantopoulos and Winklhofer (2001) provided a set of recommendations for indicator selection. Their recommendation to use multiple-indicators multiple-causes (MIMIC) models for path estimation should be ignored, however, because MIMIC models are irrelevant to formative models (Lee et al., 2013; Muthén, 1989). Theory on formative models has proceeded as far as identifying when to use them and how to estimate them, but not on how to decide which indicators to use for them. One approach to selecting indicators begins with recognizing that a formatively measured variable is essentially a variable optimized to predict a set of outcomes. Because the formatively measured variable begins as the shared variance of the outcomes, its indicators’ weights reflect only the unique variance they contribute to this shared variance. Hence, their weights, and thus the formative variable they contribute to, are optimized to predict the outcomes. From this recognition, one approach to picking indicators is to choose those that are relevant to socioeconomic status (SES) and that are uniquely related to the outcomes. Hence, income and education may be relevant for some outcomes, whereas occupation and wealth may be relevant for others. A major issue with this approach is that the chosen indicators need not be a complete representation of SES but be only the set of variables that most fully account for SES’s relation to an outcome. Thus, using only predictive indicators to represent SES in a formative model could err and omit variables important for a complete representation of SES. Thus, a better approach might be to start with a set of indicators judged to represent the breadth of SES. When entered into the model, the indicators of SES from this broader set that do not uniquely predict the outcomes will receive low weights and may need to be dropped to obtain satisfactory model fit. To my knowledge, no guidelines exist for managing this tension between model fit and content validity. (Note that this logic follows that developed by Diamantopoulos & Winklhofer, 2001, for selection and retention of indicators.) ^f^Note that variables that are reflectively measured (e.g., identity, subjective SES) should be modeled as reflective indicators of SES. Bollen and Bauldry (2011) and Bainter and Bollen (2014) provided examples of how to fit formative models. van Bork et al. (in Asendorpf et al., 2016, Figure 1, bottom half, p. 308) demonstrated how to test whether formatively measured variables affect outcomes over and above their indicators. I provide an example of these two steps in the Supplemental Material available online using the *lavaan* package in R (Rosseel, 2012).

The decision tree depicted in Figure 5 aims to help researchers select a subset of socioeconomic conditions that are consistent with the researchers’ theory and feasible to measure. These goals are accomplished by asking researchers to state their theoretical model (e.g., “SES promotes longevity”) and elaborate the set of variables involved in the model so that a particular socioeconomic condition takes the place of SES (e.g., “Income promotes longevity”). The note to Figure 5 provides citations to work describing theories of individual socioeconomic conditions, and it describes issues to keep in mind while using the tree. Note that this decision tree was developed by a U.S. researcher. It may not generalize to other cultural contexts; future research should assess its cross-cultural (and cross-temporal) utility.

### How to use the decision tree

Say a group of researchers wanted to study the relationship between SES and presidential voting in the United States in 2016. In Step 1 of the decision tree, they state their hypothesis about the relationship between SES and voting. They might hypothesize that SES is positively related to the likelihood of voting Republican. In Step 2, they spell out the process creating the hypothesized relationship. They might reason that higher-SES people have more monetary resources and want to protect them, yielding a preference for antitax policies and, hence, a preference for Republican candidates, who typically support these policies. However, after further consideration, the researchers might additionally propose that higher-SES individuals could vote for Democratic candidates because of the liberalizing effects of education. These steps correspond to the usual steps in a research project in which the hypothesis under investigation is described (Step 1), as well as a reason for its occurrence (Step 2). In Step 3, the hypothesized process is translated into a path diagram (e.g., SES → Monetary Resources, Education → Tax Views, Social Views → Voting). This step is a formalization of Steps 1 and 2, akin to drawing a directed acyclic graph (Pearl, 2000; van der Laan & Rose, 2011).

In Step 4, the researchers list the variables one step away from SES in the path diagram (e.g., Monetary Resources and Education from “SES → Monetary Resources, Education → Tax Views, Social Views → Voting”). This step begins the process of deciding which socioeconomic condition to focus on. In Steps 4a through 4c, the researchers refine and narrow the variables listed in Step 4 to be more easily measurable. Education is pretty specific but could be narrowed to personal education because the researchers’ theory applies to individuals’ personal experiences. “Monetary resources” is pretty unspecific but seems to refer to consumptive resources—resources (e.g., income) that could be used to aid consumption of other resources (e.g., food, health care). Given the focus on tax views, personal income would appear to be a relevant variable, although personal wealth could also work. Thus, in Step 4a, the researchers narrow “monetary resources” to personal income. Step 4b covers fitting a formative model to the individual socioeconomic conditions if the researchers wanted to focus on an effect of SES beyond its indicators instead of on individual socioeconomic conditions. In Step 4c, the researchers replace the variables listed in Step 4 with those named in Steps 4a or 4b (i.e., from “Monetary Resources, Education” to “Personal Income, Personal Education”). Finally, in Step 5, the path diagram is modified to remove SES so that the hypothesis becomes about how the selected socioeconomic conditions relate to the outcome (e.g., Personal Income, Personal Education → Tax Views, Social Views → Voting). This step formalizes the shift to a narrower variable, restricting the theoretical claims and interpretation to the level of the chosen socioeconomic conditions.

With the decision tree completed, the researchers can begin making a validity argument (e.g., “We measured personal income because it represents most people’s primary source of monetary resources and, thus, what they might be most likely to use in forming their views on taxes”). They can also proceed to identifying threats to validity for a measure of the newly focal variable, guard against these threats, and incorporate these protections into the validity argument (e.g., “To guard against deliberate misreporting of income and general memory issues, we obtained participants’ tax returns”). Thus, by shifting the targeted interpretation away from SES and toward specific socioeconomic conditions, the decision tree clarifies which variables should be measured (improving content validity) and eases identification of threats to validity by limiting threats to processes that produce error for the key variables (e.g., underreporting of very high income).

The Supplemental Material contains applications of the decision tree to the three most cited articles from the earlier literature review (Bigler et al., 2003; Hudson, 2005; Piff et al., 2010) and to one of my own articles (Antonoplis & Chen, 2021). It also contains an empirical test of the model for income, education, and voting developed above, using data from the 1972–2018 General Social Survey of U.S. adults.^7^ As expected, income and education correlated with voting in opposite directions, a result that the decision tree enables researchers to predict but that current recommendations for studying SES struggle to resolve.

## General Discussion

How should psychologists study SES? To answer this question, I reviewed current practices and recommendations for studying SES in psychology, commented on conceptual issues involved in studying SES, and described a solution to these problems. I found that current practices for studying SES were often not well justified and that current recommendations for studying SES produced contradictory predictions. Examining two prospective solutions to measuring SES—formative measurement models and subjective SES—I found that neither delivered a measure that could be validly interpreted as measuring SES. To resolve these issues, I proposed an alternative approach: to study individual socioeconomic conditions, such as income or education, rather than the broader SES construct. Finally, I described a decision tree to aid selection of individual socioeconomic conditions based on theory and demonstrated its utility compared with current recommendations for studying SES. Below, I contextualize these results and their implications in the broader literature and consider future directions for research.

### Toward increased theoretical integration

The reconceptualization of SES presented here is important to theoretical integration in psychology in two ways. First, it synthesizes developments in measurement theory (Bollen & Lennox, 1991; Edwards & Bagozzi, 2000; Rhemtulla et al., 2015), psychology (Kraus et al., 2012), sociology (Chan & Goldthorpe, 2007; Duncan, 1992; Weeden & Grusky, 2005), and public health (Braveman et al., 2005; Krieger et al., 1997; Shavers, 2007) to resolve a long-standing issue. Whereas sociology, public health, and psychology have often been integrated in the study of SES (e.g., Adler & Stewart, 2010), measurement theory has received less attention. The integration of measurement theory is especially important because it provides a framework for measuring and testing theories, key activities in empirical fields (Flake & Fried, 2020; Wilson, 2005).

Second, this reconceptualization helps reorganize results in the psychological literature on SES to potentially increase their policy relevance. Viewing socioeconomic conditions as individual constructs rather than as exchangeable indicators permits reorganizing results so that the effects of individual socioeconomic conditions (e.g., income, education, and occupational prestige) can be grouped across studies. Given that intervention studies target individual socioeconomic conditions—for instance, employment (e.g., transitional employment programs; Verma et al., 2005; Williams & Hendra, 2018), income (e.g., earned income tax credits in Miller et al., 2018; cash transfers in Haushofer & Shapiro, 2016; Miller et al., 2016), and education (e.g., tuition-free education; Dynarski et al., 2018; Knechtel et al., 2017)—reorganizing psychological results to focus on socioeconomic conditions moves results closer to the level at which socioeconomic interventions and policies are made.

### Toward improving measurement in psychology

Importantly, this article should be read in the context of larger discussions about measurement in psychology. As Flake et al. (2017) showed, problems similar to those discussed here are common in social-personality psychology. Likewise, clinical psychology has seen discussion of problems in cross-cultural assessment of depression (Chentsova-Dutton et al., 2007) and problems in defining depression (Borsboom & Cramer, 2013; Fried & Nesse, 2015). Political psychology has seen challenges to the meaning of ideological identity (liberalism–conservatism) in terms of its dimensionality between and within persons (Morgan & Wisneski, 2017), as well as its meaning for Black Americans (Jefferson, 2020). The present work adds to these conversations, further highlighting the importance of careful construct and measurement development.

### Future directions for the decision tree and measuring socioeconomic conditions

Finally, if future work seeks to expand the decision tree, there are three key areas for expansion: (a) incorporating theories about socioeconomic conditions, (b) incorporating information on data sources for socioeconomic conditions, and (c) adding guidance on estimation strategies. Presently, the decision tree helps researchers only after they have articulated a theoretical model linking socioeconomic conditions to an outcome. The decision tree does not help researchers choose whether material resources or cultural learning best explains an outcome for a particular population, for example, but only whether income or education would be an appropriate feature based on their theory. Likewise, the decision tree does not help researchers decide how to word items or choose between various sources of data (e.g., self-reported vs. tax-derived income) or how to statistically link predictors to outcomes. Of course, these selective focuses were deliberate, but work addressing these areas could be incorporated into future versions of the decision tree.

For instance, several overviews of the meaning of various socioeconomic conditions to health outcomes exist in public health (e.g., Galobardes et al., 2006a, 2006b; Krieger et al., 1997; Shavers, 2007) and could be added to the decision tree and modified to map the relevance of socioeconomic conditions to psychological outcomes. Likewise, Galobardes et al. (2006a, 2006b) and Braveman et al. (2005) provided templates for thinking about how age and race, respectively, alter the relevance of socioeconomic conditions to health outcomes. Future versions of the decision tree could include prompts to consider how socioeconomic conditions are relevant to particular populations under study. Bright et al. (2016) described one way quantitative social scientists might adapt the decision tree to study such hypotheses. Future versions of the decision tree might also incorporate Diemer et al.’s (2013) excellent guide for wording items and choosing data sources for the measurement of socioeconomic conditions. Finally, as one reviewer suggested, two degrees of freedom (2DF) joint tests of significance may be used when collinearity among socioeconomic conditions creates concern about tests of unique effects being underpowered (e.g., Fernández-Rhodes et al., 2016).

### Conclusion

Socioeconomic conditions are widely considered important causal factors in people’s lives (National Academies of Sciences, Engineering, and Medicine, 2019). Despite this importance, how to appropriately study and measure them has not been resolved. I developed a novel approach to conceptualizing and measuring socioeconomic conditions that seeks to better align theoretical aims with operationalizations. Thus, this article took a first step toward understanding the many ways in which socioeconomic conditions impact people’s lives in contemporary societies.

## Supplemental Material

sj-docx-1-pps-10.1177_17456916221093615 – Supplemental material for Studying Socioeconomic Status: Conceptual Problems and an Alternative Path Forward Click here for additional data file. Supplemental material, sj-docx-1-pps-10.1177_17456916221093615 for Studying Socioeconomic Status: Conceptual Problems and an Alternative Path Forward by Stephen Antonoplis in Perspectives on Psychological Science

## Transparency

*Action Editor:* Laura A. King

*Editor:* Laura A. King

*Author Contributions*

S. Antonoplis conceived the project and all study designs, analyzed data, and drafted the manuscript. S. Antonoplis is the sole author of this article and is responsible for its content.

## Notes

1. Throughout this article, I use “socioeconomic status” and “social class” interchangeably, as is the norm in psychology. Others have argued they should be used in reference to distinct concepts (Krieger et al., 1997), but given that psychologists are accustomed to equating these terms, I use them interchangeably.

2. This result, along with the correlation between education and voting for Hillary Clinton, is reported in the Supplemental Material available online, using data from the 1972–2018 General Social Surveys (Smith et al., 2018).

3. I also reviewed the relevance of four notions of composite modeling to SES and found none relevant (see Supplemental Material).

4. A realist stance on features of SES seems plausible, however. Given societal consensus on the meaning of money, income, wealth, education, occupations, and so on, researchers can say that they are real by virtue of their having been created through clear social processes (Hacking, 1999).

5. For an example of the limited contexts in which formative models can be applied, see Wilson and Gochyyev (2020). The authors referred to these contexts as “administrative,” in contrast to the usual “scientific” contexts that concern researchers, in that the contexts do not involve testing a scientific theory (pp. 5–6).

6. Of course, these arguments are not a problem if subjective SES is interpreted as a question of identity rather than a direct readout of people’s environments.

7. I thank Claude S. Fischer (personal communication, June 2019) for pointing me to this example.
