# Supplementary material for: Studying Socioeconomic Status: Conceptual Problems and an Alternative Path Forward
Source: Perspect Psychol Sci. 2022 Aug 18;18(2):275–92. doi: 10.1177/17456916221093615 (PMC10018062; doi:10.1177/17456916221093615)
Supplement: sj-docx-1-pps-10.1177_17456916221093615 – Supplemental material for Studying Socioeconomic Status: Conceptual Problems and an Alternative Path Forward [file sj-docx-1-pps-10.1177_17456916221093615.docx]

Supplemental Materials for

“Studying Socioeconomic Status: Conceptual Problems and an Alternative Path Forward”

Stephen Antonoplis

University of California, Berkeley

**Literature Review of Current Practices for Studying SES: Results (Continued)**

**Reasons for Choices of Indicators and Modeling Procedures.** How did research psychologists justify their measurement procedures? Table S1 reports the reasons for choices of indicators and modeling procedures used across studies. Many studies did not report why particular indicators (48.8%) were chosen, and most studies did not report why particular modeling procedures (84.4%) were chosen. The most common reasons for choice of indicators were that prior research reported a relationship between the indicator and the focal (or related) outcomes (17.6%) or because the indicator was (commonly) used in prior research (13.5%). Less common reasons included that the indicator was provided in the dataset used (2%), the indicator was hypothesized to relate to the outcome (2%), and that the indicator was more temporally stable than other indicators (2%). The most common reasons for choice of modeling procedure were that indicators were correlated, justifying aggregation (6.9%); that indicators represented distinct pathways from SES to the outcome, justifying not aggregating them (2.8%); and that prior research had used the same modeling procedure (2.3%). Less common reasons included that aggregating indicators maximized predictive ability relative to single indicators (0.5%) and that the modeling procedure was more appropriate for the sample size available (0.5%).

What do these trends mean for the validity of these measures of SES? About half of the indicators were not justified, making it hard to assess their validity. For the indicators that were justified, it seems that researchers have often used weak reasons to justify their choices. For instance, that an indicator is known to relate to the outcome might be defensible when SES is controlled for or used to replicate a known effect of SES. But, that a particular indicator was chosen because of its relation to an outcome suggests some indicators were not chosen due to their lack of relation to the outcome. It does seem slightly problematic to design a measurement procedure, even unintentionally, such that it guarantees (or is thought to guarantee) a result for some outcome. Similarly, that prior work used a particular procedure does not guarantee the meaningfulness or validity of the procedure. And that only certain indicators were available in a dataset does not imply that the available indicators provided a good test of one’s theory.

For modeling procedures, most procedures were not justified, making it hard to know how they are valid. For the procedures that were justified, the reasons shown in Table S1 are a mix of more and less defensible arguments. For instance, that indicators are noninterchangeable or represent distinct pathways from SES to an outcome seems defensible given it is often the case that different indicators relate to outcomes in different ways (e.g., Twenge & Campbell, 2002; Tan et al., 2020) for different groups (Braveman et al., 2005) and that theorists have begun to describe why these differential associations occur (Krieger et al., 1997; Shavers, 2007; Tan et al., 2020). In contrast, it is not clear why two indicators of SES being correlated justifies combining them. High correlations can indicate problems of multicollinearity (Mansfield & Helms, 1982), but multicollinearity can be formally tested via variance inflation factors, which none of the studies using this reason reported. From a measurement standpoint, correlations between indicators can be viewed as a result of their sharing a common cause. However, SES does not seem to cause people to have more income, education, etc., so much as it seems to result from people’s having more income, education, etc. Indeed, recent theory views SES as the result of income, education, and other factors, rather than as the cause of them (Kraus et al., 2012). Thus, it is not clear why two indicators of SES being correlated justifies combining them. Similarly, that prior research modeled indicators in a particular way is not reason alone to continue modeling them that way. Changes in the broader environment (e.g., new populations, historical changes) may necessitate alterations to older ways of modeling indicators (Hollingshead, 1971). Overall, many measurement procedures of SES were not justified and those that were had a mix of more and less defensible reasons.

| Table S1 |  |  |  |  |  |  |
| --- | --- | --- | --- | --- | --- | --- |
|  |  |  |  |  |  |  |
| *Reasons for Choice of Indicators and Modeling Procedures for SES* | | | | | | |
|  |  |  |  |  |  |  |
| Reasons |  | Percentage |  | Number (n=244) |  | Example |
|  |  |  |  |  |  |  |
| *Indicators* |  |  |  |  |  |  |
|  |  |  |  |  |  |  |
| None given |  | 48.80 |  | 119 |  | – |
|  |  |  |  |  |  |  |
| Content validity |  | 5.80 |  | 14 |  | Material resources (income) and subjective rank (the MacArthur ladder) reflect core aspects of SES |
|  |  |  |  |  |  |  |
| Data availability |  | 2.50 |  | 6 |  | Community partners blocked collection of individual-level SES indicators so used data on the schools that individuals attended |
|  |  |  |  |  |  |  |
| Dataset provided |  | 2.00 |  | 5 |  | Only education and subjective status/rank available in both datasets used (MIDUS, MIDJA) |
|  |  |  |  |  |  |  |
| Evidence based |  | 17.60 |  | 43 |  | Income predicts focal outcome (and related outcomes) better than other indicators of SES (e.g., education) |
|  |  |  |  |  |  |  |
| Parallel forms |  | 2.90 |  | 7 |  | Hollingshead’s index correlates (highly) with other indicators of SES |
|  |  |  |  |  |  |  |
| Population appropriate |  | 2.90 |  | 7 |  | Family structural assets better indexes stratification than other indicators of SES in the population studied |
|  |  |  |  |  |  |  |
| Presumed relation |  | 2.00 |  | 5 |  | Parental education and occupation, as well as personal income, thought to relate to outcome (participation in socially deviant peer groups) |
|  |  |  |  |  |  |  |
| Prior work |  | 13.50 |  | 33 |  | Hollingshead’s index is a crude but commonly used indicator in past research |
|  |  |  |  |  |  |  |
| Temporal stability |  | 2.00 |  | 5 |  | Parental education more temporally stable than other indicators (e.g., family income) |
|  |  |  |  |  |  |  |
|  |  | Percentage |  | Number (n=218) |  | Example |
|  |  |  |  |  |  |  |
| *Modeling Procedure* |  |  |  |  |  |  |
|  |  |  |  |  |  |  |
| None given |  | 84.40 |  | 184 |  | – |
|  |  |  |  |  |  |  |
| Correlated |  | 6.90 |  | 15 |  | Aggregating income, education, and subjective rank (MacArthur ladder) justified because the three were positively correlated |
|  |  |  |  |  |  |  |
| Distinct pathways |  | 2.80 |  | 6 |  | Not aggregating education, income, subjective class identification, job characteristics, and supervising experience justified because they represent distinct ways SES relates to outcome (self- vs. other-orientation) |
|  |  |  |  |  |  |  |
| Maximize prediction |  | 0.50 |  | 1 |  | Aggregating education, single motherhood, reception of welfare, poverty status, employment status, and whether the household included more than four minors improves prediction (of any outcome) relative to use of single indicators |
|  |  |  |  |  |  |  |
| Noninterchangeable |  | 0.90 |  | 2 |  | Aggregating income-to-needs ratio, perceived economic hardship, feelings about economic situation, and financial cutbacks made due to need not justified because indicators represent different aspects of SES |
|  |  |  |  |  |  |  |
| Prior work |  | 2.30 |  | 5 |  | Aggregating family income-to-needs ratio, maternal education, and maternal perceived neighborhood chaos justified because follows existing guidelines for SES measurement |
|  |  |  |  |  |  |  |
| Reduce measurement error |  | 0.90 |  | 2 |  | ZIP code median income, median education, and median occupational status modeled as a reflective latent variable (using SEM) because doing so accounts for measurement error in latent SES variable |
|  |  |  |  |  |  |  |
| Sample size |  | 0.50 |  | 1 |  | Partial least squares (PLS) works better with smaller sample sizes than other modeling techniques (e.g., SEM) |
|  |  |  |  |  |  |  |
| Unidimensional |  | 0.90 |  | 2 |  | Aggregating perceived childhood and current financial difficulty justified because the items followed a unidimensional factor model |
|  |  |  |  |  |  |  |
| *Note.* Percentages were obtained by dividing by the total number of reasons given within each category (e.g., counts of indicators  were divided by the total number of reasons given for indicators, n=244, across all studies), rather than the number of studies.  Examples quotes were paraphrased to make them more succinct. The full quotes are given in Table S2 in the Supplement. | | | | | | |

**Full Quotes of Reasons for Choices of Indicators and Modeling Procedures**

| Table S2 |  |  |  |  |  |  |
| --- | --- | --- | --- | --- | --- | --- |
|  |  |  |  |  |  |  |
| *Reasons for Choice of Indicators and Modeling Procedures for SES* | | | | | | |
|  |  |  |  |  |  |  |
| Reasons |  | Percentage |  | Number (n=243) |  | Example |
|  |  |  |  |  |  |  |
| *Indicators* |  |  |  |  |  |  |
|  |  |  |  |  |  |  |
| None given |  | 48.60 |  | 118 |  | – |
|  |  |  |  |  |  |  |
| Content validity |  | 5.80 |  | 14 |  | “We tested our hypothesis with measures of social class reflecting the two core aspects of the construct: objective indicators of material resources (e.g., income; Oakes & Rossi, 2003) and subjective perceptions of one’s social class rank in society (e.g., Adler et al., 2000).” |
|  |  |  |  |  |  |  |
| Data availability |  | 2.50 |  | 6 |  | “The reluctance of school authorities to allow questions regarding children’s family circumstances and their experience of conflict resulted in the use of less than optimal methods. For this reason, SES was indexed on the basis of the school a child attended, and only a limited number of conflict-related experiences were included in the scale administered.” |
|  |  |  |  |  |  |  |
| Dataset provided |  | 2.10 |  | 5 |  | “In Study 1, we used educational attainment and subjective social status as indices of SES because those were the only indices of SES available for both MIDUS and MIDJA.” |
|  |  |  |  |  |  |  |
| Evidence based |  | 17.70 |  | 43 |  | “In our studies, we operationally defined social class according to income, as income information is commonly reported and may predict social-class outcomes better than some other contributing factors (such as education; e.g., Côté et al., 2017).” |
|  |  |  |  |  |  |  |
| Parallel forms |  | 2.90 |  | 7 |  | “Hollingshead’s operationalization of socioeconomic status using four factors is the most widely used and validated measure of socioeconomic status in psychological research, though it is not without limitations (Cirino et al., 2002; Ribas, Moura, Soares, Gomes, & Bornstein, 2003; Suzuki & Patricoski, 2013). The measure correlates highly with other indices of social status” |
|  |  |  |  |  |  |  |
| Population appropriate |  | 2.90 |  | 7 |  | “Our focus on family structural assets as a culturally appropriate measure of disadvantage may be a better proxy of socioeconomic risks that undermine maternal opportunities to develop verbal skills than of acute stressors known to affect maternal fluid intelligence and executive function skills.” |
|  |  |  |  |  |  |  |
| Presumed relation |  | 2.10 |  | 5 |  | “The relatively stable contextual factors of family-of-origin SES (education and occupation) and income were tested because they were hypothesized to relate to ongoing developmental contextual risk for the youth.” |
|  |  |  |  |  |  |  |
| Prior work |  | 13.60 |  | 33 |  | “Although the Hollingshead SES index has been criticized for being too crude a measure (Duncan & Magnuson, 2003), it is nevertheless the best known and most widely used measure available (Bornstein et al., 2003), and therefore suitable for the aim of this study—that is, establishing direct evidence of the link between children’s SES and brain processes.” |
|  |  |  |  |  |  |  |
| Temporal stability |  | 2.10 |  | 5 |  | “Socioeconomic status was measured using residential parent’s mean level of educational attainment. Educational attainment is a commonly used index of socioeconomic status (Bradley & Corwyn, 2002), which might be more stable than family income (U.S. Treasury Department, 2008) and has been used in previous G X E analyses (e.g., Harden, Turkheimer, & Loehlin, 2007). Educational attainment was coded on a 9-point ordinal scale ranging from eighth grade or less to professional training beyond a 4-year degree.” |
|  |  |  |  |  |  |  |
|  |  | Percentage |  | Number (n=217) |  | Example |
|  |  |  |  |  |  |  |
| *Modeling Procedure* |  |  |  |  |  |  |
|  |  |  |  |  |  |  |
| None given |  | 84.80 |  | 184 |  | – |
|  |  |  |  |  |  |  |
| Correlated |  | 6.50 |  | 14 |  | “Because the three SES measures were positively correlated (r’s=.39 to .53, p’s<.01), we computed a composite score of SES for further analysis by averaging the three standardized measures (M=.02, SD=.81).” |
|  |  |  |  |  |  |  |
| Distinct pathways |  | 2.80 |  | 6 |  | “However, SES can be indexed by a wide range of variables, including material wealth and occupational conditions. Because World Values Survey (WVS) offered these SES indices, we explored how the patterns of the results might differ across different indices of SES by testing multiple facets of SES in Study 2a.” |
|  |  |  |  |  |  |  |
| Maximize prediction |  | 0.50 |  | 1 |  | “It is widely recognized that risk factors such as poverty, sin- gle parenthood, low parental education, and unemployment often occur together (Masten et al., 1995) and are generally more predictive when examined cumulatively” |
|  |  |  |  |  |  |  |
| Noninterchangeable |  | 0.90 |  | 2 |  | “Objective and subjective measures of monetary/wealth resources,economic hardship, class/status, education, and community-level variables (e.g., poverty rates, crime rates) have been used as markers of SES, yet different indicators of SES are not considered interchangeable (Braveman et al., 2005). The measures one chooses to examine SES reflect different underlying conceptualizations that may be associated with different pathways linking SES to children’s health (Bradley & Corwyn, 2002). Thus, the use of multiple indices of SES is imperative for delineating relations between SES and child health.” |
|  |  |  |  |  |  |  |
| Prior work |  | 2.30 |  | 5 |  | “In order to create an index of socioeconomic risk, we followed previous guidelines (Conger et al., 2010; Dearing, McCartney, & Taylor, 2001) and created a composite variable comprised of a family income-to-needs ratio,maternal education, and maternal report of level of chaos in the neighborhood” |
|  |  |  |  |  |  |  |
| Reduce measurement error |  | 0.90 |  | 2 |  | “SEM allows the simultaneous test of a hypothesized structure of relationships among variables of interest as well an inclusive test of the measurement model of the latent or unobserved variables (i.e., SES, mental illness). When this is not done, measurement error in the composite variables is ignored. The failure in many studies to disattenuate the correlations obscures the relationships among the major indicators.” |
|  |  |  |  |  |  |  |
| Sample size |  | 0.50 |  | 1 |  | “Partial least squares modeling is ideal for examining all of the possible associations between the variables of interest as it can be used with small data sets” |
|  |  |  |  |  |  |  |
| Unidimensional |  | 0.90 |  | 2 |  | “A principal-axis factor analysis using varimax rotation of the five social class items yielded a single factor with an eigenvalue above 1.0 (2.99) that accounted for 60% of the variance. All factor loadings exceeded .58. Moreover, the measures for childhood social class (M=4.66,SD=1.39, α=.77) and current social class (M=4.61,SD=1.58, α=.77) were highly correlated, r(112)=.58,p<.01, and the five items demonstrated high internal consistency (.82). Thus, we formed a composite measure by averaging the five childhood and current social class items (M=4.64, SD=1.29).” |
|  |  |  |  |  |  |  |
| *Note.* Percentages were obtained by dividing by the total number of reasons given within each category (e.g., counts of indicators  were divided by the total number of reasons given for indicators, n=243, across all studies), rather than the number of studies. | | | | | | |

**Is Composite Modeling Relevant to SES? Arguments Against Four Notions of *Composite***

Table S3 below describes four notions of composite in the measurement literature, as well as why they are not relevant to modeling Socioeconomic Status. One notion includes indicators more for convenience than for measuring a theoretical variable and was thus deemed unsuitable for measurement by its describers (Bollen & Bauldry, 2011; Bollen & Diamantopoulos, 2017). A second notion assumes unit-weighting for the first notion, creating further problems by biasing estimates of the composite’s relations to other variables (Bollen & Bauldry, 2011). A third notion includes measurement error from components in the definition of the composite, leading its describers to deem it inappropriate for research, where separating construct and error variance is critical (Wilson & Gochyyev, 2020). Finally, Classical Test Theory’s notion of composite is tautological and, thus, provides no test of theory, which is problematic in a research setting (Lord & Novick, 1968).

| Table S3 | | | |
| --- | --- | --- | --- |
| *Notions of “Composite”* | | | |
| Reference(s) | Semantic Definition (Summarized) | Formal Definition | Relevance to Socioeconomic Status |
| 1. Bollen & Bauldry (2011); Bollen & Diamantopoulos (2017) | Composite as weighted sum of indicators, which, in general, have no conceptual unity. | C_i_=∑w_i_x_i_ | No relevance to SES because SES is a theoretical variable measured by indicators with conceptual unity. |
| 2. Bollen & Bauldry (2011) | Scale (Index) as unit-weighted composite. | C_i_=∑1x_i_ | No relevance to SES because lack of conceptual unity, plus will produce biased estimates if weights differ from structural paths. |
| 3. Wilson & Gochyyev (2020) | Composite as superordinate domain emerging from components and that is useful for practice/administrative work, but not research. | P(X_pik_=1; A,B,ξ\|θ_p_) = exp[b’_pik_ θ_p_ + a’_ik_ ξ]/∑exp[b’_pik_ θ_p_ + a’_ik_ ξ]  θ*_p_ = **W**θ^(d)^_p_ + ε_p_ | No relevance to SES because researchers need more precise estimates that deal with measurement error better. |
| 4. Lord & Novick (1968), Ch. 5 | Composite as the aggregate of all components. | Y_i_ = T_i_ + E_i_  X = ∑Y_i_ | No relevance to SES because research tests a theory and Classical Test Theory (CTT) is tautological. As CTT cannot fail, it is a poor test of theory. |

**Example Code for Fitting a Formative Model in *lavaan***

This code is also available on OSF at <https://osf.io/tgj28/?view_only=cbfa7ca3d4cf4ecdb2ebe49b4aedd397>.

##########################################################
# goal = make example of formative measurement for paper #
##########################################################
## First, we need to simulate the data
### We'll assume that all variable are normally distributed, with normally distibued error terms
#### 0. Set up basic parameters
 set.seed(252) # for reproducibility
 nobs <- 1000 # sample 1000 "participants"
#### 1. Make a set of background variables, W, that causal indicators depend on (alternative is to simulate as multivariate normal)
 W1 <- rnorm(n=nobs,mean=0,sd=1)
 W2 <- rnorm(n=nobs,mean=0,sd=1)
 W3 <- rnorm(n=nobs,mean=0,sd=1)
 W4 <- rnorm(n=nobs,mean=0,sd=1)
 W5 <- rnorm(n=nobs,mean=0,sd=1)
#### 2. Make set of causal indicators, X, each of which depends on 3 background variables with ES=.2
 # which combinations of W for each X?
 count <- 0
 while(count < 5) {print(sample(1:5,3,replace=F)); count <- count + 1}

## [1] 3 5 1
## [1] 3 1 2
## [1] 3 2 5
## [1] 2 4 5
## [1] 4 1 3

# make X
 X1 <- .2*W3 + .2*W5 + .2*W1 + rnorm(nobs,0,.5) # make sd smaller so X depend on W more
 X2 <- .2*W3 + .2*W1 + .2*W2 + rnorm(nobs,0,.5)
 X3 <- .2*W3 + .2*W2 + .2*W5 + rnorm(nobs,0,.5)
 X4 <- .2*W2 + .2*W4 + .2*W5 + rnorm(nobs,0,.5)
 X5 <- .2*W3 + .2*W1 + .2*W3 + rnorm(nobs,0,.5)
#### 3. Make Outcomes, which depend on X, and their indicators Y and Z
 # which ß for X
 count <- 0
 while(count < 2) {print(sample(seq(from=.2,to=.6,by=.01),5,replace=F)); count <- count + 1}

## [1] 0.30 0.34 0.29 0.38 0.43
## [1] 0.59 0.30 0.54 0.58 0.50

# make Outcomes
 O1 <- .3*X1 + .34*X2 + .29*X3 + .38*X4 + .43*X5 + rnorm(nobs,0,1)
 O2 <- .59*X1 + .3*X2 + .54*X3 + .58*X4 + .5*X5 + rnorm(nobs,0,1)
 # make Y indicators for O1
 Y1 <- .8*O1 + rnorm(nobs,0,.5) # make the sd smaller so more of variance is O
 Y2 <- .8*O1 + rnorm(nobs,0,.5)
 Y3 <- .8*O1 + rnorm(nobs,0,.5)
 Y4 <- .8*O1 + rnorm(nobs,0,.5)
 Y5 <- .8*O1 + rnorm(nobs,0,.5)
 Y6 <- .8*O1 + rnorm(nobs,0,.5)
 Y7 <- .8*O1 + rnorm(nobs,0,.5)
 Y8 <- .8*O1 + rnorm(nobs,0,.5)
 # make Z indicators for O2
 Z1 <- .8*O2 + rnorm(nobs,0,.5)
 Z2 <- .8*O2 + rnorm(nobs,0,.5)
 Z3 <- .8*O2 + rnorm(nobs,0,.5)
 Z4 <- .8*O2 + rnorm(nobs,0,.5)
 Z5 <- .8*O2 + rnorm(nobs,0,.5)
 Z6 <- .8*O2 + rnorm(nobs,0,.5)
 Z7 <- .8*O2 + rnorm(nobs,0,.5)
 Z8 <- .8*O2 + rnorm(nobs,0,.5)
#### 4. Move all variable to a dataframe, and now, we can examine the correlation matrix
 df <- data.frame(X1,X2,X3,X4,X5,Y1,Y2,Y3,Y4,Y5,Y6,Y7,Y8,Z1,Z2,Z3,Z4,Z5,Z6,Z7,Z8)
 round(cov(df),2) # X somewhat correlated with each other; all X correlated with Y and Z; all Y and Z highly correlated

## X1 X2 X3 X4 X5 Y1 Y2 Y3 Y4 Y5 Y6 Y7 Y8 Z1 Z2
## X1 0.36 0.08 0.07 0.03 0.12 0.20 0.18 0.17 0.18 0.20 0.18 0.18 0.17 0.23 0.23
## X2 0.08 0.35 0.08 0.02 0.12 0.19 0.20 0.20 0.19 0.20 0.19 0.19 0.18 0.21 0.22
## X3 0.07 0.08 0.38 0.06 0.06 0.18 0.20 0.19 0.18 0.20 0.18 0.20 0.18 0.24 0.24
## X4 0.03 0.02 0.06 0.37 -0.01 0.19 0.19 0.18 0.18 0.19 0.19 0.18 0.19 0.18 0.18
## X5 0.12 0.12 0.06 -0.01 0.45 0.27 0.26 0.26 0.23 0.24 0.26 0.24 0.24 0.25 0.27
## Y1 0.20 0.19 0.18 0.19 0.27 1.20 0.98 0.99 0.98 0.96 0.96 0.95 0.95 0.37 0.40
## Y2 0.18 0.20 0.20 0.19 0.26 0.98 1.22 0.98 0.98 0.94 0.98 0.94 0.97 0.36 0.39
## Y3 0.17 0.20 0.19 0.18 0.26 0.99 0.98 1.23 0.99 0.96 0.99 0.96 0.97 0.36 0.38
## Y4 0.18 0.19 0.18 0.18 0.23 0.98 0.98 0.99 1.20 0.94 0.95 0.94 0.96 0.36 0.38
## Y5 0.20 0.20 0.20 0.19 0.24 0.96 0.94 0.96 0.94 1.20 0.95 0.91 0.95 0.38 0.43
## Y6 0.18 0.19 0.18 0.19 0.26 0.96 0.98 0.99 0.95 0.95 1.22 0.94 0.97 0.37 0.38
## Y7 0.18 0.19 0.20 0.18 0.24 0.95 0.94 0.96 0.94 0.91 0.94 1.18 0.93 0.35 0.38
## Y8 0.17 0.18 0.18 0.19 0.24 0.95 0.97 0.97 0.96 0.95 0.97 0.93 1.19 0.32 0.35
## Z1 0.23 0.21 0.24 0.18 0.25 0.37 0.36 0.36 0.36 0.38 0.37 0.35 0.32 1.28 1.03
## Z2 0.23 0.22 0.24 0.18 0.27 0.40 0.39 0.38 0.38 0.43 0.38 0.38 0.35 1.03 1.29
## Z3 0.24 0.23 0.26 0.19 0.26 0.37 0.38 0.37 0.36 0.39 0.37 0.35 0.35 1.05 1.06
## Z4 0.24 0.24 0.23 0.18 0.27 0.40 0.39 0.38 0.37 0.41 0.36 0.38 0.36 1.05 1.05
## Z5 0.23 0.22 0.26 0.19 0.25 0.41 0.40 0.41 0.37 0.42 0.39 0.39 0.37 1.02 1.01
## Z6 0.23 0.22 0.25 0.19 0.26 0.37 0.39 0.36 0.35 0.38 0.35 0.36 0.34 1.05 1.07
## Z7 0.23 0.23 0.25 0.17 0.27 0.37 0.38 0.39 0.35 0.40 0.36 0.38 0.35 1.03 1.02
## Z8 0.23 0.21 0.24 0.18 0.26 0.38 0.38 0.37 0.37 0.41 0.36 0.37 0.35 1.05 1.03
## Z3 Z4 Z5 Z6 Z7 Z8
## X1 0.24 0.24 0.23 0.23 0.23 0.23
## X2 0.23 0.24 0.22 0.22 0.23 0.21
## X3 0.26 0.23 0.26 0.25 0.25 0.24
## X4 0.19 0.18 0.19 0.19 0.17 0.18
## X5 0.26 0.27 0.25 0.26 0.27 0.26
## Y1 0.37 0.40 0.41 0.37 0.37 0.38
## Y2 0.38 0.39 0.40 0.39 0.38 0.38
## Y3 0.37 0.38 0.41 0.36 0.39 0.37
## Y4 0.36 0.37 0.37 0.35 0.35 0.37
## Y5 0.39 0.41 0.42 0.38 0.40 0.41
## Y6 0.37 0.36 0.39 0.35 0.36 0.36
## Y7 0.35 0.38 0.39 0.36 0.38 0.37
## Y8 0.35 0.36 0.37 0.34 0.35 0.35
## Z1 1.05 1.05 1.02 1.05 1.03 1.05
## Z2 1.06 1.05 1.01 1.07 1.02 1.03
## Z3 1.31 1.06 1.03 1.06 1.04 1.05
## Z4 1.06 1.29 1.02 1.05 1.03 1.03
## Z5 1.03 1.02 1.25 1.04 1.00 1.02
## Z6 1.06 1.05 1.04 1.33 1.03 1.04
## Z7 1.04 1.03 1.00 1.03 1.26 1.02
## Z8 1.05 1.03 1.02 1.04 1.02 1.28

## Now, we can fit the model
 library(lavaan)

## This is lavaan 0.6-6

## lavaan is BETA software! Please report any bugs.

library(semPlot)

## Registered S3 methods overwritten by 'huge':
## method from
## plot.sim BDgraph
## print.sim BDgraph

model_measurement <- '
 # formative portion
 SES <~ 1*X1 + X2 + X3 + X4 + X5 # indicates that X cause SES, the latent variable; first variable must be fixed to estimate rest
 # need to account for correlations between X
 X1 ~~ X2 + X3 + X4 + X5
 X2 ~~ X3 + X4 + X5
 X3 ~~ X4 + X5
 X4 ~~ X5
 # see here for other ways of fitting formative variables in lavaan (e.g., using phantom variables) https://groups.google.com/d/msg/lavaan/hnMCckaARoo/ewImVWLwLxAJ
 # reflective portion
 Outcome1 =~ Y1 + Y2 + Y2 + Y3 + Y4 + Y5 + Y6 + Y6 + Y7 + Y8
 Outcome2 =~ Z1 + Z2 + Z2 + Z3 + Z4 + Z5 + Z6 + Z6 + Z7 + Z8
 # assume unidimensionality for Y, so its residuals are uncorrelated
 # make outcomes uncorrelated
 #Outcome1 ~~ 0*Outcome2
 # hypothesis test
 #SES =~ Outcome1 + Outcome2 # this line fits the model like a MIMIC model
 Outcome1 ~ SES # just standard regression; SES will not be identified without this step
 Outcome2 ~ SES
 '
 mm_sem <- sem(model_measurement,data=df)
 summary(mm_sem,standardized=T,fit.measures=T)

## lavaan 0.6-6 ended normally after 65 iterations
##
## Estimator ML
## Optimization method NLMINB
## Number of free parameters 54
##
## Number of observations 1000
##
## Model Test User Model:
##
## Test statistic 198.760
## Degrees of freedom 177
## P-value (Chi-square) 0.126
##
## Model Test Baseline Model:
##
## Test statistic 20246.777
## Degrees of freedom 210
## P-value 0.000
##
## User Model versus Baseline Model:
##
## Comparative Fit Index (CFI) 0.999
## Tucker-Lewis Index (TLI) 0.999
##
## Loglikelihood and Information Criteria:
##
## Loglikelihood user model (H0) -19106.349
## Loglikelihood unrestricted model (H1) -19006.969
##
## Akaike (AIC) 38320.698
## Bayesian (BIC) 38585.717
## Sample-size adjusted Bayesian (BIC) 38414.209
##
## Root Mean Square Error of Approximation:
##
## RMSEA 0.011
## 90 Percent confidence interval - lower 0.000
## 90 Percent confidence interval - upper 0.018
## P-value RMSEA <= 0.05 1.000
##
## Standardized Root Mean Square Residual:
##
## SRMR 0.016
##
## Parameter Estimates:
##
## Standard errors Standard
## Information Expected
## Information saturated (h1) model Structured
##
## Latent Variables:
## Estimate Std.Err z-value P(>|z|) Std.lv Std.all
## Outcome1 =~
## Y1 1.000 0.988 0.900
## Y2 1.002 0.022 44.684 0.000 0.989 0.896
## Y3 1.011 0.022 45.156 0.000 0.998 0.899
## Y4 0.995 0.022 44.903 0.000 0.983 0.897
## Y5 0.975 0.023 42.714 0.000 0.963 0.880
## Y6 0.995 0.023 44.077 0.000 0.983 0.891
## Y7 0.968 0.023 42.668 0.000 0.955 0.879
## Y8 0.986 0.022 44.412 0.000 0.974 0.893
## Outcome2 =~
## Z1 1.000 1.021 0.903
## Z2 0.999 0.022 45.637 0.000 1.020 0.899
## Z3 1.012 0.022 46.246 0.000 1.033 0.904
## Z4 1.002 0.022 46.102 0.000 1.023 0.903
## Z5 0.979 0.022 44.890 0.000 1.000 0.894
## Z6 1.008 0.023 44.630 0.000 1.029 0.892
## Z7 0.983 0.022 44.793 0.000 1.003 0.893
## Z8 0.994 0.022 45.325 0.000 1.015 0.897
##
## Composites:
## Estimate Std.Err z-value P(>|z|) Std.lv Std.all
## SES <~
## X1 1.000 0.502 0.300
## X2 0.977 0.159 6.137 0.000 0.491 0.292
## X3 1.085 0.164 6.625 0.000 0.545 0.334
## X4 1.360 0.183 7.450 0.000 0.683 0.416
## X5 1.215 0.182 6.684 0.000 0.610 0.410
##
## Regressions:
## Estimate Std.Err z-value P(>|z|) Std.lv Std.all
## Outcome1 ~
## SES 0.286 0.033 8.773 0.000 0.578 0.578
## Outcome2 ~
## SES 0.324 0.036 8.955 0.000 0.632 0.632
##
## Covariances:
## Estimate Std.Err z-value P(>|z|) Std.lv Std.all
## X1 ~~
## X2 0.076 0.011 6.591 0.000 0.076 0.213
## X3 0.065 0.012 5.531 0.000 0.065 0.178
## X4 0.026 0.012 2.258 0.024 0.026 0.072
## X5 0.121 0.013 9.166 0.000 0.121 0.303
## X2 ~~
## X3 0.080 0.012 6.739 0.000 0.080 0.218
## X4 0.020 0.011 1.729 0.084 0.020 0.055
## X5 0.120 0.013 9.074 0.000 0.120 0.300
## X3 ~~
## X4 0.064 0.012 5.340 0.000 0.064 0.171
## X5 0.063 0.013 4.809 0.000 0.063 0.154
## X4 ~~
## X5 -0.013 0.013 -0.995 0.320 -0.013 -0.031
## .Outcome1 ~~
## .Outcome2 0.010 0.021 0.455 0.649 0.015 0.015
##
## Variances:
## Estimate Std.Err z-value P(>|z|) Std.lv Std.all
## .Y1 0.228 0.012 19.324 0.000 0.228 0.189
## .Y2 0.241 0.012 19.486 0.000 0.241 0.198
## .Y3 0.236 0.012 19.362 0.000 0.236 0.191
## .Y4 0.234 0.012 19.429 0.000 0.234 0.195
## .Y5 0.271 0.014 19.940 0.000 0.271 0.226
## .Y6 0.251 0.013 19.636 0.000 0.251 0.206
## .Y7 0.268 0.013 19.950 0.000 0.268 0.227
## .Y8 0.240 0.012 19.555 0.000 0.240 0.202
## .Z1 0.235 0.012 19.419 0.000 0.235 0.184
## .Z2 0.246 0.013 19.563 0.000 0.246 0.191
## .Z3 0.240 0.012 19.410 0.000 0.240 0.183
## .Z4 0.238 0.012 19.447 0.000 0.238 0.185
## .Z5 0.252 0.013 19.739 0.000 0.252 0.202
## .Z6 0.274 0.014 19.796 0.000 0.274 0.205
## .Z7 0.256 0.013 19.760 0.000 0.256 0.203
## .Z8 0.251 0.013 19.638 0.000 0.251 0.196
## X1 0.356 0.016 22.361 0.000 0.356 1.000
## X2 0.354 0.016 22.361 0.000 0.354 1.000
## X3 0.377 0.017 22.361 0.000 0.377 1.000
## X4 0.370 0.017 22.361 0.000 0.370 1.000
## X5 0.452 0.020 22.361 0.000 0.452 1.000
## SES 0.000 0.000 0.000
## .Outcome1 0.650 0.036 18.141 0.000 0.666 0.666
## .Outcome2 0.626 0.034 18.205 0.000 0.601 0.601

semPaths(mm_sem,layout='tree',what='std',rotation=2)


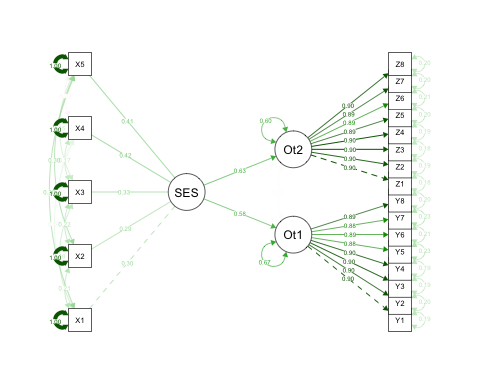


## And let's modify the model to test that SES impact O independently of its indicators
 model_test <- '
 # formative portion
 SES <~ 1*X1 + 1*X2 + 1*X3 + 1*X4 + 1*X5 # indicates that X cause SES, the latent variable; fix all paths so model will be identified
 # need to account for correlations between X
 X1 ~~ X2 + X3 + X4 + X5
 X2 ~~ X3 + X4 + X5
 X3 ~~ X4 + X5
 X4 ~~ X5
 # see here for other ways of fitting formative variables in lavaan (e.g., using phantom variables) https://groups.google.com/d/msg/lavaan/hnMCckaARoo/ewImVWLwLxAJ
 # reflective portion
 Outcome1 =~ Y1 + Y2 + Y2 + Y3 + Y4 + Y5 + Y6 + Y6 + Y7 + Y8
 Outcome2 =~ Z1 + Z2 + Z2 + Z3 + Z4 + Z5 + Z6 + Z6 + Z7 + Z8
 # assume unidimensionality for Y, so its residuals are uncorrelated
 # make outcomes uncorrelated
 Outcome1 ~~ 0*Outcome2
 # hypothesis test
 Outcome1 ~ SES + X1 + X2 + X3 + X4 # can add up to K-1 predictors in this example, where K is the total number of predictors
 Outcome2 ~ SES + X1 + X2 + X3 + X4
 '
 mt_sem <- sem(model_test,data=df)
 summary(mt_sem,standardized=T,fit.measures=T)

## lavaan 0.6-6 ended normally after 56 iterations
##
## Estimator ML
## Optimization method NLMINB
## Number of free parameters 57
##
## Number of observations 1000
##
## Model Test User Model:
##
## Test statistic 192.374
## Degrees of freedom 174
## P-value (Chi-square) 0.162
##
## Model Test Baseline Model:
##
## Test statistic 20246.777
## Degrees of freedom 210
## P-value 0.000
##
## User Model versus Baseline Model:
##
## Comparative Fit Index (CFI) 0.999
## Tucker-Lewis Index (TLI) 0.999
##
## Loglikelihood and Information Criteria:
##
## Loglikelihood user model (H0) -19103.156
## Loglikelihood unrestricted model (H1) -19006.969
##
## Akaike (AIC) 38320.312
## Bayesian (BIC) 38600.054
## Sample-size adjusted Bayesian (BIC) 38419.019
##
## Root Mean Square Error of Approximation:
##
## RMSEA 0.010
## 90 Percent confidence interval - lower 0.000
## 90 Percent confidence interval - upper 0.018
## P-value RMSEA <= 0.05 1.000
##
## Standardized Root Mean Square Residual:
##
## SRMR 0.014
##
## Parameter Estimates:
##
## Standard errors Standard
## Information Expected
## Information saturated (h1) model Structured
##
## Latent Variables:
## Estimate Std.Err z-value P(>|z|) Std.lv Std.all
## Outcome1 =~
## Y1 1.000 0.988 0.900
## Y2 1.002 0.022 44.690 0.000 0.989 0.896
## Y3 1.011 0.022 45.165 0.000 0.998 0.899
## Y4 0.995 0.022 44.901 0.000 0.983 0.897
## Y5 0.975 0.023 42.709 0.000 0.963 0.879
## Y6 0.995 0.023 44.090 0.000 0.983 0.891
## Y7 0.967 0.023 42.661 0.000 0.955 0.879
## Y8 0.986 0.022 44.425 0.000 0.974 0.894
## Outcome2 =~
## Z1 1.000 1.021 0.903
## Z2 0.999 0.022 45.634 0.000 1.020 0.899
## Z3 1.012 0.022 46.257 0.000 1.034 0.904
## Z4 1.002 0.022 46.104 0.000 1.023 0.903
## Z5 0.979 0.022 44.896 0.000 1.000 0.894
## Z6 1.008 0.023 44.632 0.000 1.029 0.892
## Z7 0.983 0.022 44.799 0.000 1.003 0.893
## Z8 0.994 0.022 45.325 0.000 1.015 0.897
##
## Composites:
## Estimate Std.Err z-value P(>|z|) Std.lv Std.all
## SES <~
## X1 1.000 0.563 0.336
## X2 1.000 0.563 0.335
## X3 1.000 0.563 0.346
## X4 1.000 0.563 0.343
## X5 1.000 0.563 0.379
##
## Regressions:
## Estimate Std.Err z-value P(>|z|) Std.lv Std.all
## Outcome1 ~
## SES 0.395 0.043 9.260 0.000 0.710 0.710
## X1 -0.155 0.070 -2.196 0.028 -0.156 -0.093
## X2 -0.117 0.070 -1.664 0.096 -0.118 -0.071
## X3 -0.133 0.064 -2.095 0.036 -0.135 -0.083
## X4 0.044 0.058 0.757 0.449 0.045 0.027
## Outcome2 ~
## SES 0.354 0.042 8.463 0.000 0.616 0.616
## X1 0.009 0.069 0.136 0.892 0.009 0.006
## X2 -0.035 0.069 -0.512 0.609 -0.035 -0.021
## X3 0.040 0.063 0.636 0.524 0.039 0.024
## X4 0.045 0.057 0.787 0.431 0.044 0.027
##
## Covariances:
## Estimate Std.Err z-value P(>|z|) Std.lv Std.all
## X1 ~~
## X2 0.076 0.011 6.591 0.000 0.076 0.213
## X3 0.065 0.012 5.531 0.000 0.065 0.178
## X4 0.026 0.012 2.258 0.024 0.026 0.072
## X5 0.121 0.013 9.166 0.000 0.121 0.303
## X2 ~~
## X3 0.080 0.012 6.739 0.000 0.080 0.218
## X4 0.020 0.011 1.729 0.084 0.020 0.055
## X5 0.120 0.013 9.074 0.000 0.120 0.300
## X3 ~~
## X4 0.064 0.012 5.340 0.000 0.064 0.171
## X5 0.063 0.013 4.809 0.000 0.063 0.154
## X4 ~~
## X5 -0.013 0.013 -0.995 0.320 -0.013 -0.031
## .Outcome1 ~~
## .Outcome2 0.000 0.000 0.000
##
## Variances:
## Estimate Std.Err z-value P(>|z|) Std.lv Std.all
## .Y1 0.228 0.012 19.324 0.000 0.228 0.189
## .Y2 0.241 0.012 19.486 0.000 0.241 0.198
## .Y3 0.236 0.012 19.362 0.000 0.236 0.191
## .Y4 0.234 0.012 19.432 0.000 0.234 0.195
## .Y5 0.272 0.014 19.943 0.000 0.272 0.227
## .Y6 0.251 0.013 19.635 0.000 0.251 0.206
## .Y7 0.268 0.013 19.953 0.000 0.268 0.227
## .Y8 0.239 0.012 19.553 0.000 0.239 0.202
## .Z1 0.235 0.012 19.419 0.000 0.235 0.184
## .Z2 0.246 0.013 19.565 0.000 0.246 0.192
## .Z3 0.240 0.012 19.408 0.000 0.240 0.183
## .Z4 0.238 0.012 19.448 0.000 0.238 0.185
## .Z5 0.252 0.013 19.738 0.000 0.252 0.202
## .Z6 0.274 0.014 19.797 0.000 0.274 0.205
## .Z7 0.256 0.013 19.760 0.000 0.256 0.203
## .Z8 0.251 0.013 19.639 0.000 0.251 0.196
## X1 0.356 0.016 22.361 0.000 0.356 1.000
## X2 0.354 0.016 22.361 0.000 0.354 1.000
## X3 0.377 0.017 22.361 0.000 0.377 1.000
## X4 0.370 0.017 22.361 0.000 0.370 1.000
## X5 0.452 0.020 22.361 0.000 0.452 1.000
## SES 0.000 0.000 0.000
## .Outcome1 0.647 0.036 18.140 0.000 0.664 0.664
## .Outcome2 0.624 0.034 18.204 0.000 0.599 0.599

semPaths(mt_sem,layout='tree',what='std',rotation=2)


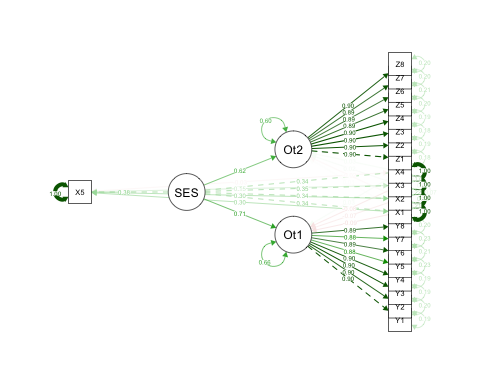


**Examples of Using the Decision Tree to Study Socioeconomic Conditions**

A few examples may help illustrate how to use the decision tree. The first example comes from one of my own articles; the next three come from the most cited articles in the literature review in the manuscript.

***1. Antonoplis & Chen (2020)*.** Antonoplis and Chen (2020) investigated whether SES impacted how people thought about and valued their future self. Studies 1a, 1b, and 2 used a composite index of income, education, and two subjective indicators. Study 3 manipulated income as a proxy for SES. Figure S1 shows the decision tree applied to their theoretical model (with quotations from the article).

Briefly narrating Figure S1: Antonoplis and Chen hypothesized that lower-SES individuals would think about and value their future selves less than higher-SES individuals (*Step 1*) because of differences in access to stable and larger sources of income, wealth, and other monetary resources (*Step 2*). Within a path diagram (*Step 3*), these monetary resources followed immediately from SES (*Step 4*), all were measurable (*Step 4a*), and no broader effect of SES above the indicators was desired (*Step 4b*). Hence, the narrower socioeconomic variables were easily added to the path diagram (*Step 4c*), and the revised path diagram highlighted that the project’s focus should be narrowed to these monetary factors (*Step 5*).

Given that this article is already published, how should its results be interpreted in light of the decision tree? The article included results for one of the appropriate socioeconomic variables, personal income. Hence, the results presented in this article should be interpreted only for personal income. Instead of being a paper about SES, then, this article presents results suggesting that lower-income people tend to think about and value their future selves less than high-income people.


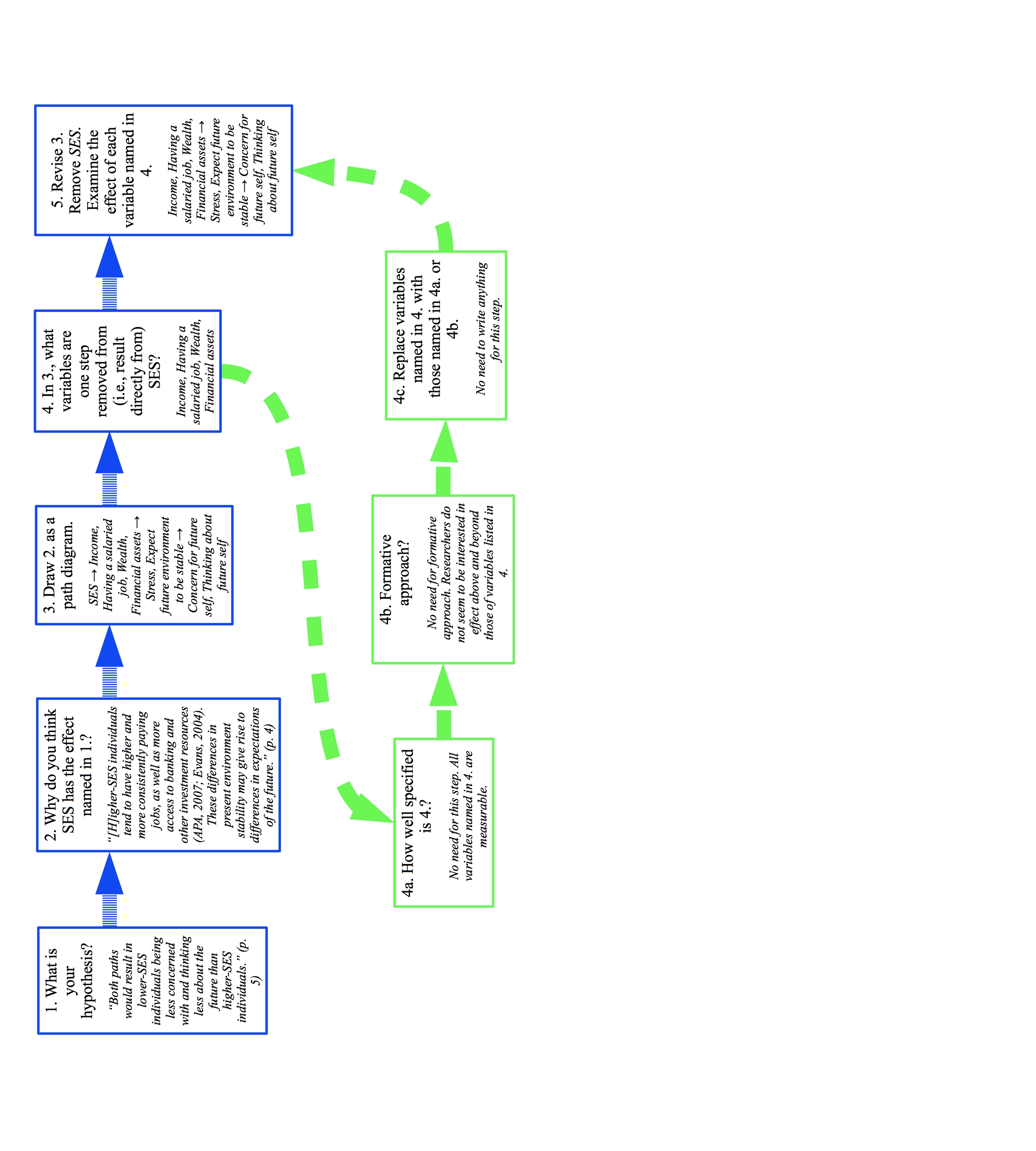


*Figure S1*

***2. Piff, Kraus, Coté, Cheng, and Keltner (2010)*.** Piff et al. (2010) investigated whether SES impacted prosocial behavior such that lower-SES individuals engaged in more prosocial behavior than higher-SES individuals. Study 1 measured SES as the MacArthur Ladder relative to participants’ communities. Study 2 manipulated participants’ perceived position on the MacArthur Ladder relative to the U.S. Study 3 used a composite of education and income. Study 4 used a composite of childhood and current annual income. Figure S2 shows the decision tree applied to their theoretical model.

Briefly narrating Figure S2: Piff et al. hypothesized that lower-SES individuals would act more pro-socially than higher-SES individuals (*Step 1*) because of differences in access to material wealth that created dependence on others and, therefore, more concern for others’ welfare (*Step 2*). Within a path diagram (*Step 3*), material wealth followed immediately from SES (*Step 4*), was measurable (*Step 4a*), and no broader effect of SES above the indicators seemed desired (*Step 4b*). Hence, the narrower socioeconomic variables were easily added to the path diagram (*Step 4c*), and the revised path diagram highlighted that the project’s focus should be narrowed to material wealth (*Step 5*).

Given that this article is already published, how should its results be interpreted in light of the decision tree? The measures and manipulations of SES used in the studies included subjective SES, current income, childhood income, and education. None of these is wealth (wealth = assets - debts; Diemer et al., 2013). Thus, it is not immediately clear how the results presented in this article pertain to the effects of material wealth on pro-sociality. Income gets a little bit close to wealth, and ratings of subjective SES may be based on wealth. Still, the compositing of current income with education (Study 3) and childhood income (Study 4) makes the results for current income alone difficult to know, and subjective SES ratings include more than wealth (Adler & Stewart, 2007). Hence, it is not entirely clear what this article says about the effect of material wealth on pro-social behavior.


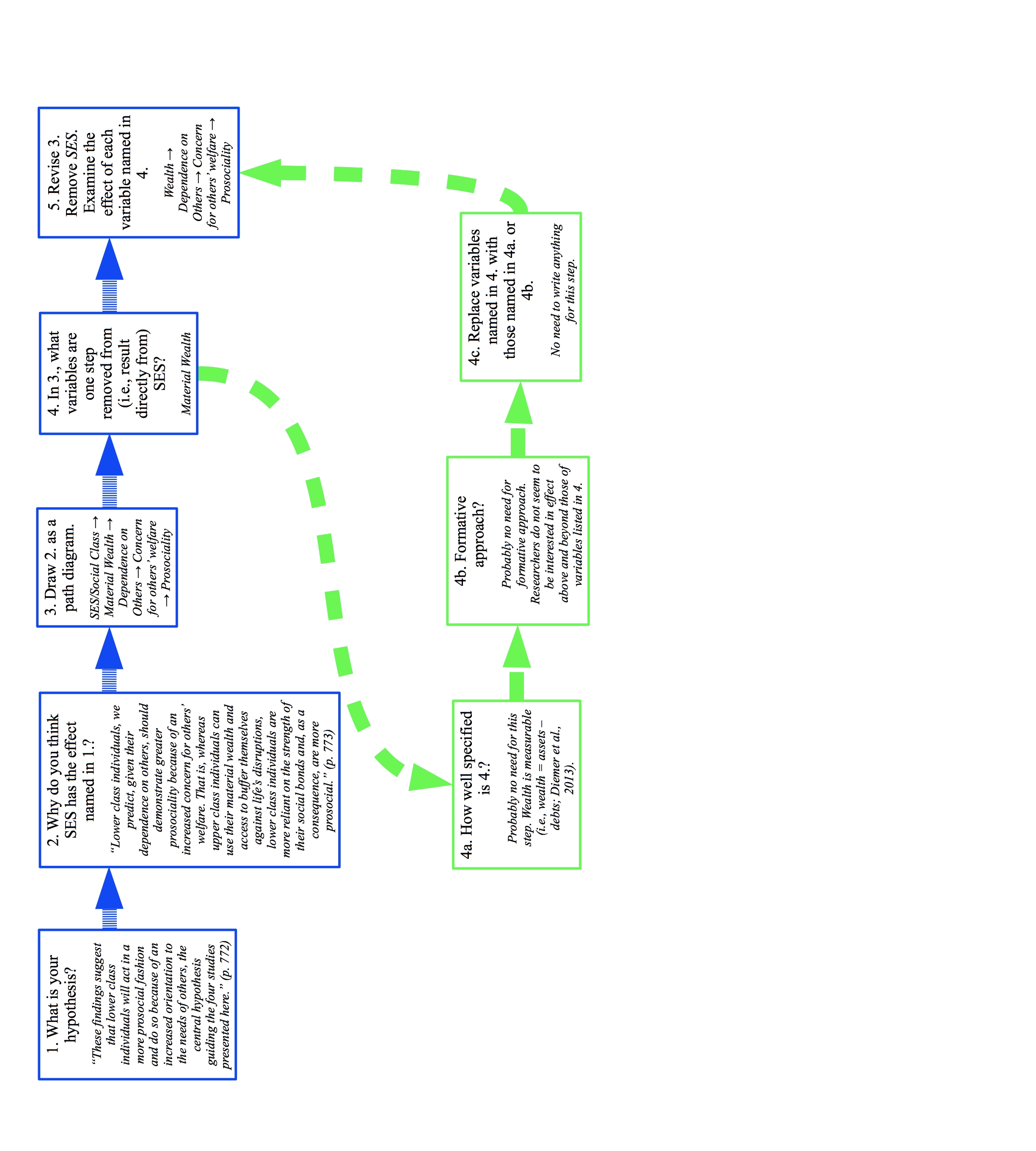


*Figure S2*

***3.*** ***Hudson (2005)*.** Hudson (2005) examined two explanations of the negative correlation between SES and mental health: a.) that occupying a lower-SES environment causes worse mental health (*social causation hypothesis*) and b.) that people experiencing worse mental health experience difficulty maintaining jobs and achieving higher education, restricting their access to income (*social selection hypothesis*). Supporting the social causation hypothesis, Hudson found that community SES, measured reflectively through ZIP-code average income, occupation, and education, significantly predicted worse mental health, mediated by community-level experience of economic hardship (measured reflectively through poverty rate, cost of housing, and unemployment). Figure S3 shows the decision tree applied to part a.) of his model.

Briefly narrating Figure S3: Hudson hypothesized that lower-SES individuals would experience more mental illness (*Step 1*) because of stressful economic conditions (*Step 2*). Within a path diagram (*Step 3*), stressful economic conditions followed immediately from SES (*Step 4*), was measurable (*Step 4a*), and a broader effect of SES above the indicators seemed desired (*Step 4b*). Hence, a formative approach in which indicators of SES and stressful economic conditions caused these higher-order constructs was recommended, as it it not totally clear how ZIP codes possess either of these constructs in a way that causes community-level outcomes, whereas it seems reasonable to say that the fact that lower-income people live somewhere makes it a low-income neighborhood. The formative variables were added to the path diagram (*Step 4c*), and the revised path diagram highlighted that the project’s focus should be on how SES and stressful economic conditions cause mental illness above and beyond their indicators (*Step 5*).

Given that this article is already published, how should its results be interpreted in light of the decision tree? The current analyses do not provide information about the new focus given by the decision tree, but the data could be re-analyzed to reflect the decision tree. Conditional on the results not changing in the new analyses, the main changes to the interpretation of the results would focus on what SES is. Instead of systematic differences in communities’ SES and economic stress causing income, education, and unemployment levels in the communities, differences in levels of indicators in the community would would give rise to differences in levels of SES and economic stress, which would together give rise to differences in levels of mental illness, beyond the differences attributable to the indicators.

*
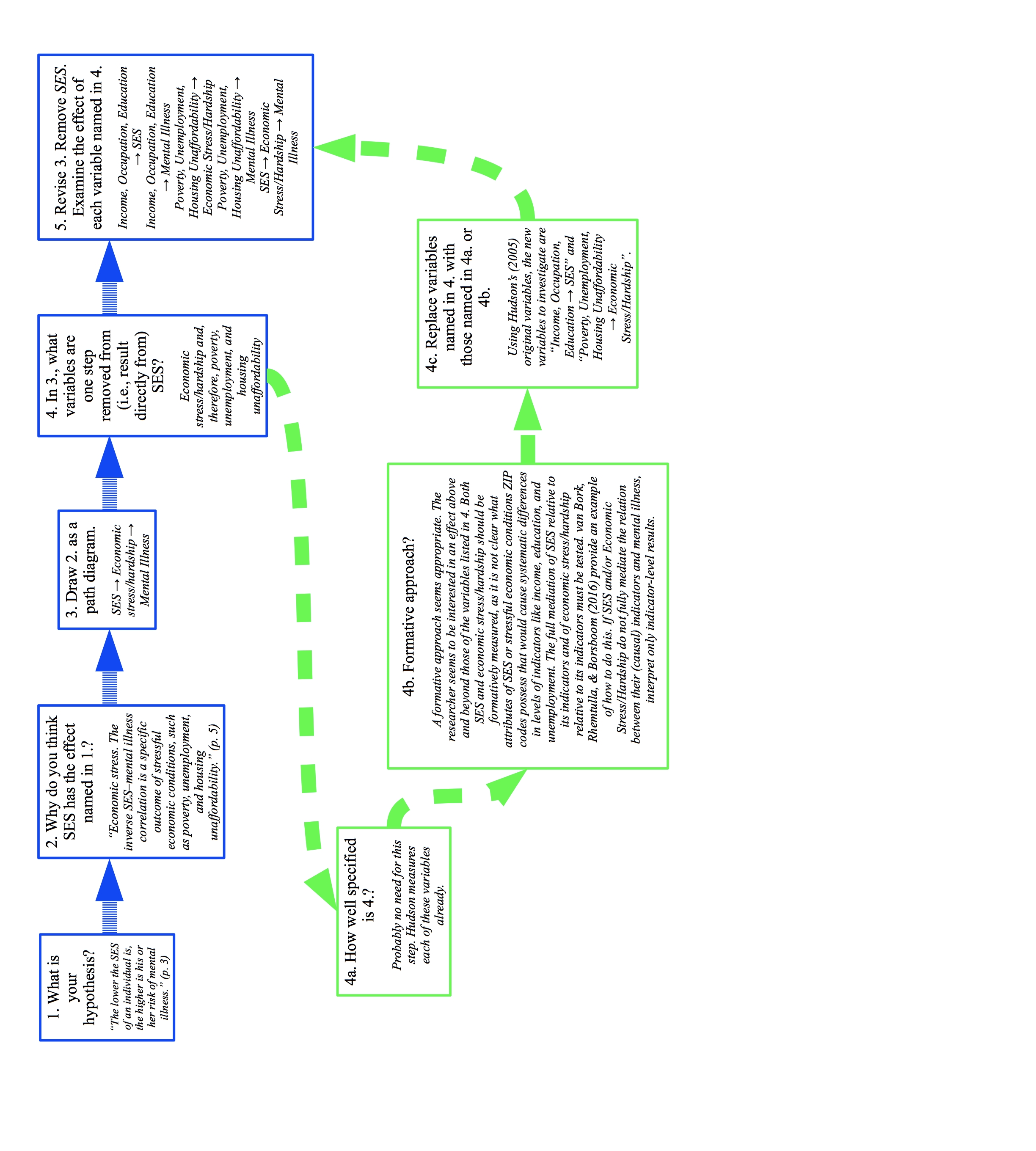
*

*Figure S3*

***4. Bigler, Averhart, & Liben (2003)*.** In an exploratory, descriptive study, Bigler et al. (2003) investigated how African American children (ages 6–7 and 11–12) from high- and low-SES backgrounds perceived the status of high-, medium-, and low-status occupations, as well as how interested they were in such jobs. They derived children’s SES from whether the children qualified for free and reduced-priced meal programs at their schools (p. 573). Qualifying for free or reduced-priced meals is based solely on parental income (USDA, 2019). Thus, Bigler and colleagues used a dichotomization of income in order to measure SES. Figure S4 shows the decision tree applied to their theoretical model.

Briefly narrating Figure S4: Bigler et al. investigated a.) the occupational stereotypes of African American children and b.) whether they held occupational aspirations in line with their parents’ SES (*Step 1*). Focusing on part b.) of Step 1, Bigler et al. hypothesized that children relied on knowledge of their parents’ occupational prestige, as determined by the parents’ SES, in forming their own aspirations (*Step 2*). Within a path diagram (*Step 3*), parental occupational prestige followed immediately from parental SES (*Step 4*), was measurable (although perhaps with some difficulty; *Step 4a*), and a broader effect of SES above the indicator did not seem desired (*Step 4b*). Hence, the narrower socioeconomic variables were easily added to the path diagram (*Step 4c*), and the revised path diagram highlighted that the project’s focus should be narrowed to parental occupational prestige (*Step 5*).

Given that this article is already published, how should its results be interpreted in light of the decision tree? The use of parental income to represent parental occupational prestige and, therefore, SES is not an uncomplicated procedure. Income might adequately proxy occupational prestige, although I found that in the General Social Survey, a nationally representative survey in the U.S., they correlated only *r*=.33 pooling across all data from 1972–2018 (*min*=.14, *max*=.42). Hence, income might not adequately proxy occupational prestige. If so, this makes Bigler et al.’s results somewhat difficult to interpret in terms of learned aspirations from parental occupational prestige.

*
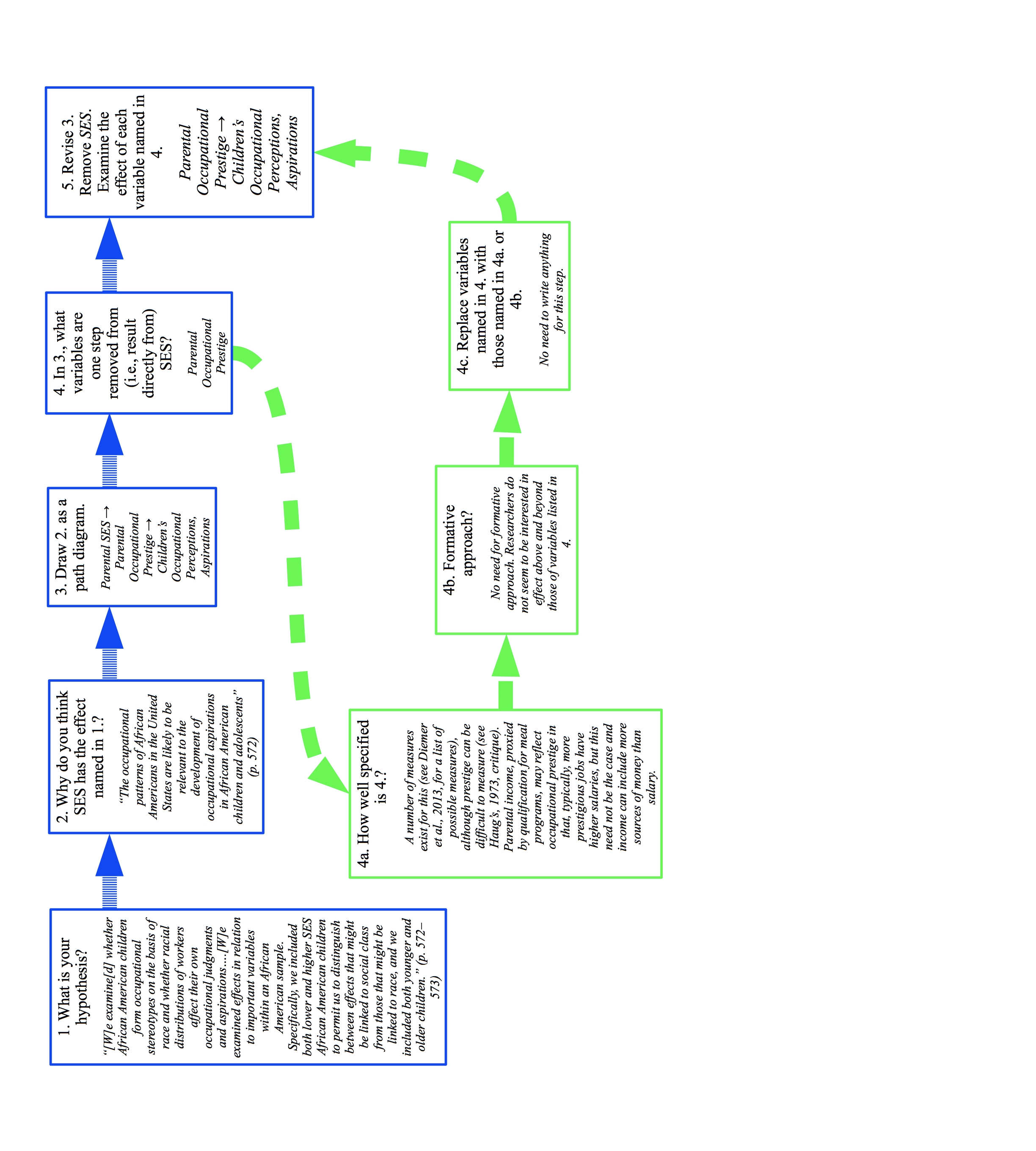
*

*Figure S4*

**An Advantage of the Decision Tree: Resolving Contradictory Results**

This paper opened with three contradictory effects of SES. Higher-SES, versus lower-SES, individuals were more likely to vote for both Donald Trump and Hilary Clinton. They reported feeling both more and less stress the day before the survey. And they wanted both more and less social distance from people with mental illness. These contradictory results could all be explained by whether the studies measured SES with income or education, which related to the outcomes differently. Here, I report results for the 2016 U.S. Presidential Election. I also examine temporal changes in the relationship between education, income, and voting for Republican versus Democratic presidential candidates.

If current recommendations to use single indicators as measures of SES are useful, then following these recommendations should not yield inconsistent predictions. In contrast, if studying single indicators on their own is useful, then similar predictions should not necessarily be made for different indicators. In other words, for current recommendations to not yield inconsistent predictions, different indicators (income, education) should relate to outcomes (voting) in similar ways within years and over time. In contrast, when single indicators are allowed to have independent meaning, no expectation of similar effects needs to be held. Based on the path model developed in the previous section, I predict that more educated Americans will be more likely to vote for the Democratic candidate, Hillary Clinton, whereas higher-income Americans will be more likely to vote for the Republican candidate, Donald Trump.

***Data*.** Data were obtained from the 1972–2018 General Social Survey (GSS) of U.S. adults (all data available from the GSS; the dataset is available at <https://gssdataexplorer.norc.org/projects/69496>; Smith et al., 2018). Only the 2018 survey contained items about voting in the 2016 U.S. Presidential Election; all other years, in addition to 2018, were used to examine temporal changes in the relationship between education, income, and voting for Republican versus Democratic candidates. Voting was keyed in the direction of voting for the Republican candidate (=1) versus the Democratic candidate (=0). Education was measured by asking participants to report their highest degree obtained (results for total years of education were virtually identical and are reported in the Supplement). Income was measured by asking participants to report their personal income and then adjusting it for inflation (unadjusted income could have been used to equal effect, as adjustment does not impact rank-order within years).

***Results*.** Due to its nominal nature, correlations with voting were estimated using polychoric and polyserial correlations, using the *polycor* R package (Version 0.7-10; Fox, 2019). RCode for all analyses can be found at <https://osf.io/tgj28/?view_only=cbfa7ca3d4cf4ecdb2ebe49b4aedd397>.

Examining voting in the 2016 election, the polychoric correlation with education was -.08, *p*=.021, 95% CI [-.15,-.01]. More educated Americans were significantly more likely to vote for the Democratic candidate, Hillary Clinton. The polyserial correlation with income was .10, *p*<.001, 95% CI [.01,.18]. Higher-income Americans were significantly more likely to vote for the Republican candidate, Donald Trump.

Figure S5 shows education and income’s correlations with voting across the 1968–2016 presidential elections. Up to the 2000 election, education and income generally correlated with voting in the same direction: Both more educated and higher-income people were more likely to vote for the Republican candidate. Education and income diverged in the 2000 election, however. Income remained positively correlated wth voting for the Republican candidate after 2000; education became negatively, or not at all, correlated with voting for the Republican candidate.

| 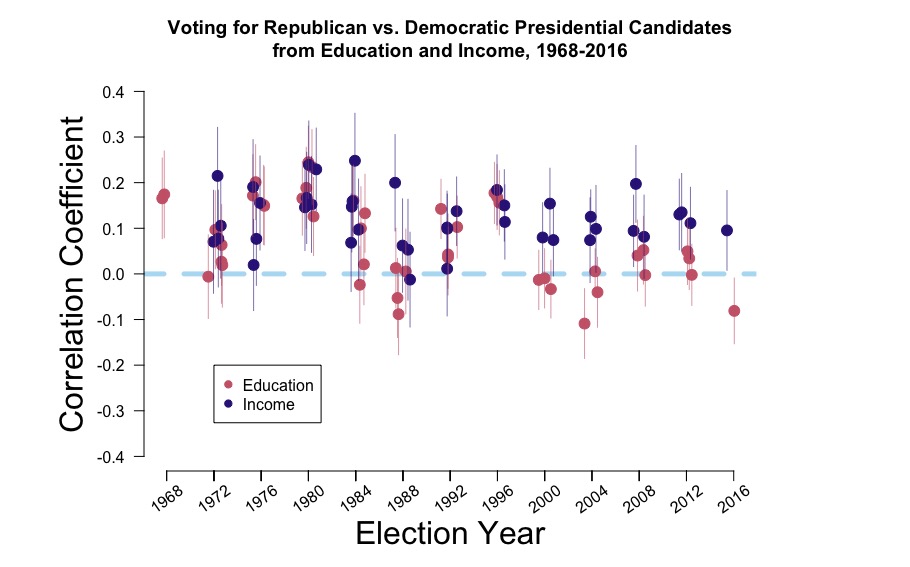 |
| --- |
| *Figure S5*. Coefficients are jittered around the year in order to enhance visual distinction between them. Multiple estimates per election comes from multiple samples querying voting for the same election. |

***Discussion.*** As predicted, more educated Americans were more likely to vote for the Democratic candidate, Hillary Clinton. In contrast, higher-income Americans were more likely to vote for the Republican candidate, Donald Trump. In addition, more educated Americans’ voting for Democratic candidates was a recent phenomenon in U.S. electoral politics. Prior to the 2000 election, more educated Americans tended to vote Republican. Higher-income Americans were more likely to vote Republican across the whole 1968–2016 period.

These results are inconsistencies that the current set of recommendations for studying SES struggles to resolve. If income and education measure the same construct, they should correlate with the same outcome in the same direction. Moreover, temporal changes in how one indicator relates to an outcome should be matched by other indicators. That income and education do not satisfy these criteria suggests that they are not merely interchangeable indicators of SES. How would the current recommendations resolve these inconsistencies? One possibility is to claim that SES does not affect voting, as the asymmetries should cancel out after aggregation. Another is to say that only income or only education truly measures SES. Neither of these seems particularly satisfactory. Both income and education relate to voting and both are widely considered measures of SES (see Table 1). The decision tree offers a satisfying solution: Income and education do not constitute measures of SES but instead describe two distinct material and social conditions that shape people’s voting behavior and that undergo distinct historical processes. This perspective fits well with recent perspectives describing the theoretical meaning of different socioeconomic conditions in general (Krieger et al., 1997; Shavers, 2007) and across the lifespan (Galobardes et al., 2006a, 2006b), as well as how the meanings of these conditions are culturally embedded (Miyamoto et al., 2018). Thus, the decision tree aids understanding of the relationship between socioeconomic conditions, behavior, and psychology.

**Correlations of Socioeconomic Conditions with Identification with Republican vs. Democratic Parties from 1972–2018**

**Correlations with Income and Education**

Whereas voting is an important behavior, identification with political parties can be an important precursor to voting (Campbell & Valen, 1961; Jackson & Carsey, 2002; Knoke, 1972; Knoke & Hout, 1974; Marshall, 2019). Thus, I also examined how socioeconomic conditions correlated with identification with the Republican vs. Democratic parties to examine whether the same patterns emerged for education relative to other variables. Due to the ordinal nature of party identification, correlations with party identification were estimated using polychoric and polyserial correlations, using the *polycor* R package, due to the ordinal nature of identification (Version 0.7-10; Fox, 2019).

I first analyzed correlations for income and education (as highest degree obtained), and Figure S6 displays the results. As with voting, education was positively related to identification as a Republican from 1972 until the 2000s, becoming near null and negatively related thereafter, particularly in the 2010s. In contrast, income correlated positively with identification as a Republican across the whole period. Thus, more educated people have gone from being more likely to identify as Republicans to being split or more likely to identify as Democrats. Higher-income people have been more likely to identify as Republicans since at least the 1970s.

If we consider both income and education measures of SES, then we need to conclude that SES is both positively and increasingly negatively correlated with identifying as a Republican. This is a paradoxical, contradictory result. If we allow income and education to separate measures of separate construct, then their opposite correlations with the same outcome are no problem and reveal potentially interesting mechanisms about how income and education impact people’s political beliefs.

| 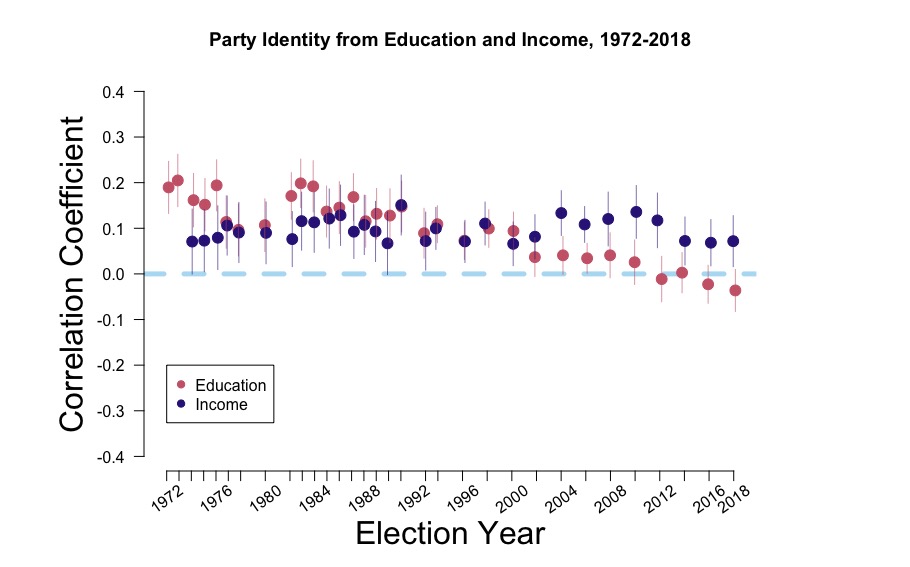 |
| --- |
| *Figure S6.* Each estimate comes from a single year (sample). Data on income was not collected in 1972 and 1973, and thus, no estimates are provided for those years. |

**References**

Adler, N. E., & Stewart, J. (2007, March). *The MacArthur Scale of Subjective Social Status*. https://macses.ucsf.edu/research/psychosocial/subjective.php

Antonoplis, S., & Chen, S. (2020). Time and class: How socioeconomic status shapes conceptions of the future self. *Self and Identity*, 1–21. https://doi.org/10.1080/15298868.2020.1789730

Bigler, R. S., Averhart, C. J., & Liben, L. S. (2003). Race and the workforce: Occupational status, aspirations, and stereotyping among African American children. *Developmental Psychology*, *39*(3), 572–580. https://doi.org/10.1037/0012-1649.39.3.572

Bollen, K. A., & Bauldry, S. (2011). Three Cs in measurement models: Causal indicators, composite indicators, and covariates. *Psychological Methods*, *16*(3), 265–284. https://doi.org/10.1037/a0024448

Bollen, K. A., & Diamantopoulos, A. (2017). In defense of causal-formative indicators: A minority report. *Psychological Methods*, *22*(3), 581–596. https://doi.org/10.1037/met0000056

Braveman, P. A., Cubbin, C., Egerter, S., Chideya, S., Marchi, K. S., Metzler, M., & Posner, S. (2005). Socioeconomic Status in Health Research: One Size Does Not Fit All. *JAMA*, *294*(22), 2879. https://doi.org/10.1001/jama.294.22.2879

Campbell, A., & Valen, H. (1961). Party Identification in Norway and the United States. *Public Opinion Quarterly*, *25*(4), 505. https://doi.org/10.1086/267047

Fox, J. (2019). *polycor: Polychoric and Polyserial Correlations* (R package version 0.7–1.0) [Computer software]. https://CRAN.R-project.org/package=polycor

Galobardes, B., Shaw, M., Lawlor, D. A., Lynch, J. W., & Smith, G. D. (2006a). Indicators of socioeconomic position (part 1). *Journal of Epidemiology & Community Health*, *60*(1), 7–12. https://doi.org/10.1136/jech.2004.023531

Galobardes, B., Shaw, M., Lawlor, D. A., Lynch, J. W., & Smith, G. D. (2006b). Indicators of socioeconomic position (part 2). *Journal of Epidemiology & Community Health*, *60*(2), 95–101. https://doi.org/10.1136/jech.2004.028092

Hollingshead, A. B. (1971). Commentary on “The Indiscriminate State of Social Class Measurement.” *Social Forces*, *49*, 563–567.

Hudson, C. G. (2005). Socioeconomic Status and Mental Illness: Tests of the Social Causation and Selection Hypotheses. *American Journal of Orthopsychiatry*, *75*(1), 3–18. https://doi.org/10.1037/0002-9432.75.1.3

Jackson, R. A., & Carsey, T. M. (2002). Group Effects on Party Identification and Party Coalitions across the United States. *American Politics Research*, *30*(1), 66–92. https://doi.org/10.1177/1532673X02030001003

Knoke, D. (1972). A Causal Model for the Political Party Preferences of American Men. *American Sociological Review*, *37*(6), 679. https://doi.org/10.2307/2093579

Knoke, D., & Hout, M. (1974). Social and Demographic Factors in American Political Party Affiliations, 1952-72. *American Sociological Review*, *39*(5), 700. https://doi.org/10.2307/2094315

Kraus, M. W., Piff, P. K., Mendoza-Denton, R., Rheinschmidt, M. L., & Keltner, D. (2012). Social class, solipsism, and contextualism: How the rich are different from the poor. *Psychological Review*, *119*(3), 546–572. https://doi.org/10.1037/a0028756

Krieger, N., Williams, D. R., & Moss, N. E. (1997). Measuring Social Class in US Public Health Research: Concepts, Methodologies, and Guidelines. *Annual Review of Public Health*, *18*(1), 341–378. https://doi.org/10.1146/annurev.publhealth.18.1.341

Lord, F. M., & Novick, M. R. (1968). *Statistical theories of mental test scores*. Addison-Wesley.

Mansfield, E. R., & Helms, B. P. (1982). Detecting Multicollinearity. *The American Statistician*, *36*(3a), 158–160. https://doi.org/10.1080/00031305.1982.10482818

Marshall, J. (2019). The Anti‐Democrat Diploma: How High School Education Decreases Support for the Democratic Party. *American Journal of Political Science*, *63*(1), 67–83. https://doi.org/10.1111/ajps.12409

Miyamoto, Y., Yoo, J., Levine, C. S., Park, J., Boylan, J. M., Sims, T., Markus, H. R., Kitayama, S., Kawakami, N., Karasawa, M., Coe, C. L., Love, G. D., & Ryff, C. D. (2018). Culture and Social Hierarchy: Self- and Other-Oriented Correlates of Socioeconomic Status Across Cultures. *Journal of Personality and Social Psychology*, *115*(3), 427–445.

Piff, P. K., Kraus, M. W., Côté, S., Cheng, B. H., & Keltner, D. (2010). Having less, giving more: The influence of social class on prosocial behavior. *Journal of Personality and Social Psychology*, *99*(5), 771–784. https://doi.org/10.1037/a0020092

Shavers, V. L. (2007). Measurement of Socioeconomic Status in Health Disparities Research. *JOURNAL OF THE NATIONAL MEDICAL ASSOCIATION*, *99*(9), 11.

Smith, T. W., Davern, M., Freese, J., & Morgan, S. (2018). *General Social Surveys, 1972-2018 [machine-readable data file]*. Chicago: NORC, 2018: NORC at the University of Chicago [producer and distributor]. Data accessed from the GSS Data Explorer website at gssdataexplorer.norc.org.

Tan, J. J. X., Kraus, M. W., Carpenter, N. C., & Adler, N. E. (2020). The association between objective and subjective socioeconomic status and subjective well-being: A meta-analytic review. *Psychological Bulletin*, *146*(11), 970–1020. https://doi.org/10.1037/bul0000258

Twenge, J. M., & Campbell, W. K. (2002). Self-Esteem and Socioeconomic Status: A Meta-Analytic Review. *Personality and Social Psychology Review*, *6*(1), 59–71. https://doi.org/10.1207/S15327957PSPR0601_3

Wilson, M., & Gochyyev, P. (2020). Having your cake and eating it too: Multiple dimensions and a composite. *Measurement*, *151*, 107247. https://doi.org/10.1016/j.measurement.2019.107247
